# The disulfide catalyst QSOX1 maintains the colon mucosal barrier by regulating Golgi glycosyltransferases

**DOI:** 10.15252/embj.2022111869

**Published:** 2022-10-17

**Authors:** Tal Ilani, Nava Reznik, Noa Yeshaya, Tal Feldman, Patrick Vilela, Zipora Lansky, Gabriel Javitt, Michal Shemesh, Ori Brenner, Yoav Elkis, Neta Varsano, Ana M Jaramillo, Christopher M Evans, Deborah Fass

**Affiliations:** ^1^ Department of Chemical and Structural Biology Weizmann Institute of Science Rehovot Israel; ^2^ Life Sciences Core Facilities Weizmann Institute of Science Rehovot Israel; ^3^ Department of Veterinary Resources Weizmann Institute of Science Rehovot Israel; ^4^ Almog Diagnostic Shoham Israel; ^5^ Department of Chemical Research Support Weizmann Institute of Science Rehovot Israel; ^6^ Department of Immunology and Microbiology, School of Medicine University of Colorado Aurora CO USA; ^7^ Department of Medicine, School of Medicine University of Colorado Aurora CO USA

**Keywords:** colon, glycosyltransferases, mucus, redox homeostasis, sulfhydryl oxidase, Digestive System, Post-translational Modifications & Proteolysis

## Abstract

Mucus is made of enormous mucin glycoproteins that polymerize by disulfide crosslinking in the Golgi apparatus. QSOX1 is a catalyst of disulfide bond formation localized to the Golgi. Both QSOX1 and mucins are highly expressed in goblet cells of mucosal tissues, leading to the hypothesis that QSOX1 catalyzes disulfide‐mediated mucin polymerization. We found that knockout mice lacking QSOX1 had impaired mucus barrier function due to production of defective mucus. However, an investigation on the molecular level revealed normal disulfide‐mediated polymerization of mucins and related glycoproteins. Instead, we detected a drastic decrease in sialic acid in the gut mucus glycome of the QSOX1 knockout mice, leading to the discovery that QSOX1 forms regulatory disulfides in Golgi glycosyltransferases. Sialylation defects in the colon are known to cause colitis in humans. Here we show that QSOX1 redox control of sialylation is essential for maintaining mucosal function.

## Graphical abstract

The Golgi‐localized enzyme QSOX1 is required for the establishment of a protective mucus layer by promoting sialic acid incorporation into mucosal glycans.

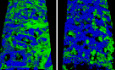

## Introduction

Gastrointestinal health depends on the successful construction and performance of protective mucus secreted by goblet cells into the gut lumen (Johansson *et al*, 2013; Melhem *et al*, 2021). Mucus is a hydrogel, composed of networks of mucin glycoproteins, that serves as a physical barrier separating pathogenic microorganisms, viruses, and parasites from the underlying epithelium (Sharpe *et al*, 2018; Wagner *et al*, 2018). Mucins also feed the symbiotic gut microbiome (Sicard *et al*, 2017) and interact with proteins that aid in tissue regeneration and repair (Braga Emidio *et al*, 2020). Some progress has been made in understanding the molecular structures of mucins (Hughes *et al*, 2019; Javitt *et al*, 2019, 2020a; Ridley *et al*, 2019), but factors that contribute to mucin functional organization and maintenance *in vivo* are poorly understood. The mucosal barrier defects associated with inflammatory bowel diseases have been suggested to arise post‐transcriptionally (Smillie *et al*, 2019), at the levels of protein synthesis, oligomerization, and post‐translational modification. An improved mechanistic understanding of mucus construction in goblet cells may offer new opportunities to strengthen weak links in the mucin scaffold and enhance the protective activity of the hydrogel, thereby improving resistance to infections and inflammation (Gouyer *et al*, 2015).

Mucin glycoproteins form long polymers linked by disulfide bonds acquired in a stepwise intracellular assembly process. First, intermolecular disulfide bonds crosslink two mucin carboxy termini to form dimers in the endoplasmic reticulum (ER; Perez‐Vilar *et al*, 1996). Subsequently, multiple mucin dimers are connected to form polymers by disulfide bonding of their amino termini in the Golgi apparatus or other post‐ER compartments (Perez‐Vilar *et al*, 1998; Perez‐Vilar & Hill, 1999). The carboxy‐terminal crosslinking (Lippok *et al*, 2016), along with extensive intramolecular disulfide bonding, is likely to be carried out by the numerous thiol‐disulfide oxidoreductases that comprise the general oxidative protein folding machinery of the ER (Appenzeller‐Herzog & Ellgaard, 2008), with the aid of specific factors such as Agr2 (Park *et al*, 2009; Zhao *et al*, 2010). In contrast, the potential players in disulfide‐mediated mucin polymerization in the Golgi are more limited. Quiescin sulfhydryl oxidase 1 (QSOX1), a catalyst of disulfide bond formation localized to the Golgi in various cell types (Ilani *et al*, 2013), is expressed at high levels in intestinal goblet cells (Tury *et al*, 2006), where mucus is produced. It was thus reasonable to speculate that QSOX1 introduces the intermolecular disulfides in the second step of mucin polymer biosynthesis. In this study, we set out to test this hypothesis.

Assays of mucin bioassembly are complicated by the same features that give these proteins their extraordinary structural and mechanical capabilities. Mucins stand out for their unusual lengths, extensive glycosylation, and large number of intramolecular disulfide bonds. For example, human intestinal mucin 2 (MUC2) is more than 5,000 amino acids long, has over 100 disulfides, and can bear up to five times its protein weight in O‐linked glycans (Strous & Dekker, 1992; Arike & Hansson, 2016). Furthermore, goblet cells are highly differentiated factories for mucin production (Nyström *et al*, 2021) that are not easily adapted to cell culture and genetic manipulation, though airway epithelial cell lines have been used successfully to study mucin mesoscale structure (Carpenter *et al*, 2021). To ensure a physiological context for studying the role of QSOX1 in mucin assembly, we generated and examined QSOX1 knockout (KO) mice (capitalized QSOX1 will be used herein for both the murine and the human orthologs), focusing our analysis on the colonic mucosa. Our findings show that QSOX1 activity is indeed related to mucus hydrogel production, but that Golgi disulfide bonding impacts mucus quality and functionality by an unanticipated pathway.

## Results

### QSOX1 localizes to the Golgi apparatus of colon epithelial cells

**Figure 1:**
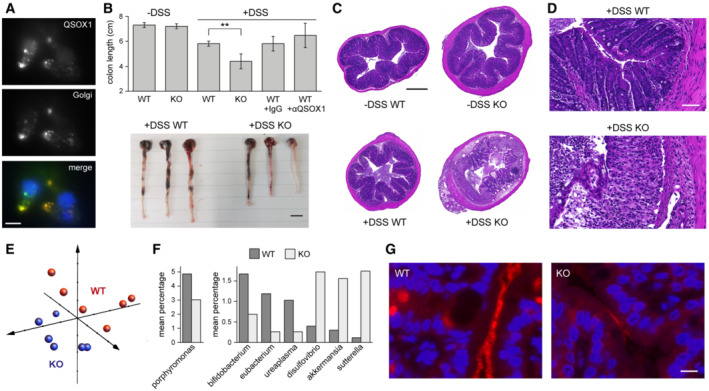
QSOX1 KO mice are hypersensitive to induced colitis and show alterations in their microbiome Immunofluorescence labeling of QSOX1 and a Golgi marker (GM130) in epithelial cells isolated from murine colon. QSOX1 is green, GM130 is red, and DAPI staining of nuclei is blue in the merged image. Scale bar is 10 μm.Top, average colon length of WT and QSOX1 KO mice following indicated treatments. For untreated mice, colon lengths were calculated for 3 WT and 3 KO. For treated mice, lengths were calculated from 5 mice except for the DSS‐treated WT, for which 3 mice were analyzed. Error bars are standard deviation. Statistical analysis was performed using Tukey multiple comparison of means (**P < 0.01). Administration of QSOX1 inhibitory antibody MAb316.1 (αQSOX1, Grossman *et al*, 2016; Feldman *et al*, 2020) did not enhance the sensitivity of WT colons to DSS (*P* > 0.4 for MAb316.1‐treated WT vs. WT control antibody (IgG)‐treated; *P* > 0.5 for MAb316.1‐treated WT vs. WT without antibody treatment). Bottom, representative images of colons following DSS treatment. Scale bar is 1 cm.Representative colon cross‐sections stained with H&E show severe damage to the epithelium in DSS‐treated KO mice. Scale bar is 500 μm.Damage to epithelium and immune cell infiltration in representative DSS‐treated QSOX1 KO colons. Scale bar is 50 μm.Principal coordinate plot of weighted Unifrac data from amplicon sequencing of feces from 5 WT and 5 KO mice using 16S universal eubacterial primers. The three vectors presented exhibit more than 70% of the variation among the groups. ANOSIM R statistic is 0.3, *P*‐value 0.011.Mean percentage is displayed for genera that differ significantly between WT and KO fecal samples and that represent at least 1% of the sequences for either genotype. This experiment was done once, with 6 mice per group.Fluorescence *in situ* hybridization of 16S rRNA (red). Blue indicates DAPI staining of nuclei. Scale bar is 10 μm. Representative images are shown from labeling of 5 WT and 5 KO mice. The experiment was performed twice on different sections from the same set of mice.

QSOX1 is expressed at high levels during development (Zanata *et al*, 2005; Portes *et al*, 2008) and in a cell‐type specific manner in particular adult tissues (Tury *et al*, 2006). Single‐cell transcriptomics data from large intestine reported by others (Tabula Muris Consortium, 2018) and confirmed by us (Appendix Fig S1) show that QSOX1 levels are highest in goblet cells. Consistent with QSOX1 intracellular localization in other cell types (Ilani *et al*, 2013), QSOX1 immunofluorescence co‐localized with a Golgi marker in cells isolated from murine colon epithelium (Fig 1A).

### QSOX1 knockout mice are highly susceptible to induced colitis and have an altered microbiome

QSOX1 KO mice were generated using commercial embryonic stem cells in a C57BL/6 background. KO mice were viable, fertile, had a normal lifespan, and showed no overt physical or behavioral abnormalities, in accordance with a previous report (Caillard *et al*, 2018). QSOX1 KO mice did not display the growth retardation, occult blood loss, and rectal prolapse exhibited by mice lacking Muc2, the major intestinal gel‐forming mucin (Van der Sluis *et al*, 2006). However, when the gastrointestinal track was challenged by administration of dextran sodium sulfate (DSS) according to an established protocol for inducing acute colitis (Okayasu *et al*, 1990; Eichele & Kharbanda, 2017), QSOX1 KO mice were found to be more sensitive, displaying severe disease symptoms (i.e., diarrhea, rectal bleeding, prolapse) on days four and five, when WT littermates were only mildly affected (i.e., no symptoms or loose stool; clinical scores were 1–2 for WT and 3–4 for KO, see Materials and Methods). After DSS exposure, colons of KO mice were shorter than those of WT, indicating more widespread epithelial loss (Fig 1B). Administration of QSOX1 inhibitory antibody (αQSOX1), which inhibits extracellular QSOX1, did not replicate this phenotype (Fig 1B). Colons of DSS‐treated KO mice showed pervasive disruption of ultrastructure (Fig 1C) and massive immune cell infiltration (Fig 1D), while colons of DSS‐treated WT mice were largely unaffected.

Though untreated WT and QSOX1 KO animals did not show appreciable differences in colon ultrastructure, the microbiome composition of co‐housed WT and KO mice clustered according to mouse genotype (Fig 1E). Bacterial genera that were elevated in KO colons include Desulfovibrio and Sutterella, previously identified as associated with active colitis (Rooks *et al*, 2014), as well as Akkermansia, which subsists on mucins (Derrien *et al*, 2004) and is considered a beneficial probiotic (Cani & de Vos, 2017; Fig 1F and Appendix Fig S2). The genera Porphyromonas, Bifidobacterium, Eubacterium, and Ureaplasma were elevated in WT mice compared to the KO (Fig 1F and Appendix Fig S2). Interestingly, fluorescence *in situ* hybridization to detect 16S rRNA showed weak signal in KO compared to WT colon sections (Fig 1G). The explanation for this difference became clear upon examining colon mucus of the QSOX1 KO animals.

### QSOX1 knockout mice have defects in colon mucus

**Figure 2:**
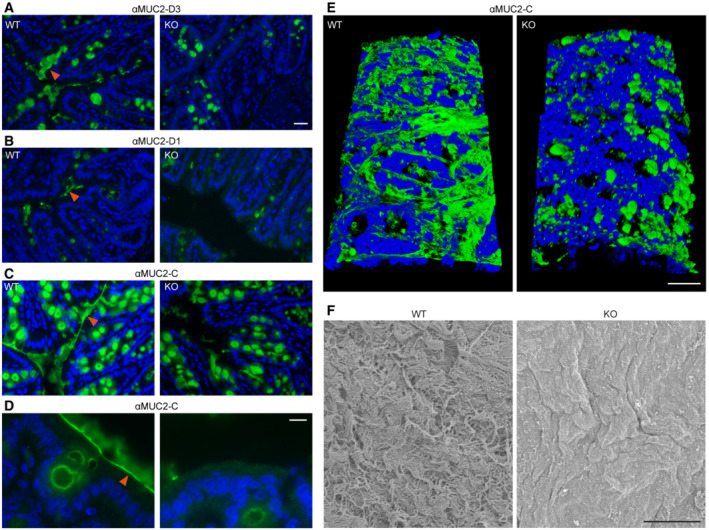
Perturbed colon mucins in QSOX1 KO mice Cross‐sections of WT and KO colons immunolabeled (green) for Muc2 with an antibody recognizing the D3 assembly in the amino‐terminal segment (αMuc2‐D3; Javitt *et al*, 2020a). Blue is DAPI staining of nuclei. Orange arrowheads indicate secreted mucin. Scale bar is 20 μm and applies also to panels (B and C).As in panel (A) except using an antibody recognizing the D1 assembly in the amino‐terminal segment (αMuc2‐D1). The antibody was raised against the human ortholog but is cross‐reactive with murine Muc2.As in panel (A) except using an antibody recognizing the Muc2 carboxy terminus (αMuc2‐C).A higher magnification image of a WT colon cross‐section shows a sharp line of Muc2 coating the epithelium (orange arrowhead), covered by diffuse mucin labeling. These features are absent from KO colons. Scale bar is 10 μm. All labeling described above was performed at least 5 times on 5 colons of each genotype. Shown are representative images.Immunolabeling (αMuc2‐C; green) of the lumen of longitudinally cut colon sections. Blue is DAPI staining of nuclei. Scale bar is 50 μm. Incomplete coverage of the colon lumen by mucin strands even in WT may be due to limitations of the fixation procedure. This experiment was done 3 times, and a representative image is shown.SEM micrographs reveal thick mucus coating a WT colon in contrast to the exposed epithelial cell surface of a KO colon. Scale bar is 5 μm. This imaging was done with one sample per genotype, and a representative image is presented.

Mucosal barrier function and bacterial colonization depend on colon mucus quantity and quality. Though transcript levels of mucin genes and factors required for mucin production and trafficking were increased in QSOX1 KO colons compared to WT colons (Appendix Fig S3), an inspection on the protein level revealed decreased Muc2 in the luminal spaces of QSOX1 KO colon cross‐sections. Lower luminal Muc2 immunofluorescence labeling was reproducible with multiple antibodies recognizing either the Muc2 amino terminus (Fig 2A and B) or carboxy terminus (Fig 2C). Most strikingly, the colons of WT mice showed a continuous, sharp line of Muc2 coating the epithelial layer, whereas the KO mice showed only diffuse and weaker extracellular Muc2 labeling (Fig 2D). A complementary perspective was obtained by a longitudinal colon cut to expose the lumen and visualize the secreted mucus from above. Examined either by immunofluorescence (Fig 2E and Appendix Fig S4) or scanning electron microscopy (SEM; Fig 2F), the lumen of WT mice showed mucin bundles covering the epithelium, contrasting with either naked, exposed cell surface or small, disorganized mucin patches in the KO. The lack of luminal mucus in fixed *ex vivo* colon samples from QSOX1 KO mice is consistent with the low 16S RNA labeling (Fig 1G), as bacteria inhabit colon mucus.

**Figure 3:**
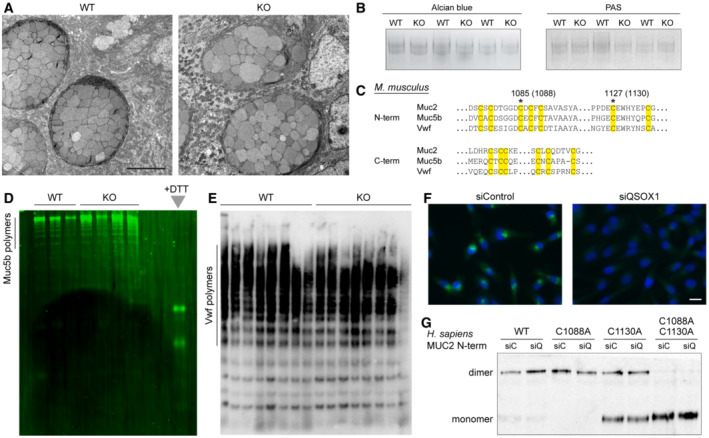
Normal mucin and Vwf polymerization in QSOX1 KO mice Representative TEM micrographs of about a hundred collected from three mice of each genotype show large and well‐packed goblet cell granules in WT and QSOX1 KO colons. Scale bar is 5 μm.Alcian blue and PAS staining of reduced guanidine‐insoluble mucins from WT and QSOX1 KO mice, separated on a 6% polyacrylamide gel.Amino acid sequences of murine Muc2 (Uniprot Q80Z19), Muc5b (Uniprot E9Q5I3), and Vwf (Uniprot Q8CIZ8) showing sequence conservation in the regions of the cysteines participating in intermolecular disulfide bonding to form polymers. Cysteines are highlighted in yellow, and asterisks indicate those that make intermolecular disulfides. Numbers indicate the amino acid positions of intermolecular disulfide‐bonding cysteines in the amino‐terminal region of murine Muc2, and in parentheses are corresponding amino acid positions in human MUC2, for reference to panel G.Muc5b fluorescent immunoblot of WT and QSOX1 KO lung lavage samples separated on agarose gels.Western blot analysis of blood Vwf from WT and QSOX1 KO mice separated on agarose gels.QSOX1 immunofluorescence (green) in control (siControl) and QSOX1 knockdown (siQSOX1) MDA‐MB‐231 cells. Blue is DAPI staining of nuclei. Scale bar is 20 μm.Western blot analysis of the MUC2 N‐terminal region and indicated cysteine mutants in supernatants of transfected cell cultures. No differences in disulfide‐mediated dimerization were observed for any MUC2 variant between siC (siControl) and siQ (siQSOX1). Data information: In panels (B, D, and E) each lane corresponds to a sample from one mouse. The results described in panels (F and G) are representative of three experiments that were performed. Source data are available online for this figure.

Downstream of the Golgi apparatus, mucins are packaged into large granules in goblet cells to await secretion. To determine whether packaging defects occur in the absence of QSOX1, transmission electron microscopy (TEM) was performed on thin colon sections. In both WT and KO animals, full mucin granules were observed (Fig 3A). Many granules in both WT and KO mice showed characteristic well‐delineated, polygonal sub‐compartments, but there seemed to be an increased number of immature granules with rounder sub‐compartments in the KO, consistent with increased mucin production and turnover rates. Together these observations establish that QSOX1 KO mice synthesize and package mucins. Upon release, however, the defect becomes clear, with the KO mice being unable to produce or retain a densely assembled mucin network (Fig 2A–E).

### Mucins and related glycoproteins polymerize in QSOX1 knockout mice

Directly evaluating polymerization of colon mucins is technically difficult due to their excessive size and the insolubility of the assembled network (Herrmann *et al*, 1999). We therefore took three complementary approaches to address this issue. First, we compared the guanidine‐insoluble fraction of colon lysates, which contains Muc2, by staining with Alcian blue and periodic acid‐Schiff (PAS). We consistently observed similar amounts of staining for WT and KO, suggesting similar amounts of reducible polymerized mucins produced by goblet cells (Fig 3B). Second, we took advantage of the fact that polymerized lung mucin Muc5b and the blood clotting glycoprotein von Willebrand factor (Vwf), which are homologous to Muc2 (Fig 3C) and share the same polymerization mechanism (Dang *et al*, 2011; Javitt *et al*, 2020a), can be analyzed by agarose gel electrophoresis. QSOX1 knockout did not prevent polymerization of either Muc5b (Fig 3D) or Vwf (Fig 3E). As a third approach to monitoring mucin disulfide bonding, we used a recombinant system comprising the amino‐terminal ~1,400 amino‐acid segment of human MUC2, which forms disulfide‐bonded dimers that represent the crosslinking step proposed to occur in the Golgi (Javitt *et al*, 2019, 2020a). When this amino‐terminal region of MUC2 was expressed in a cell line after depletion of QSOX1 (Fig 3F), similar extents of dimerization occurred as in control cells, even after sensitization of the system by eliminating either of the two intermolecular disulfide crosslinks (Javitt *et al*, 2019; Javitt *et al*, 2020a; Fig 3G). In summary, we found no evidence that mucin disulfide‐mediated polymerization is impaired in the absence of QSOX1.

### Disulfide bonding in Golgi glycosyltransferases is perturbed in QSOX1 KO mice

**Figure 4:**
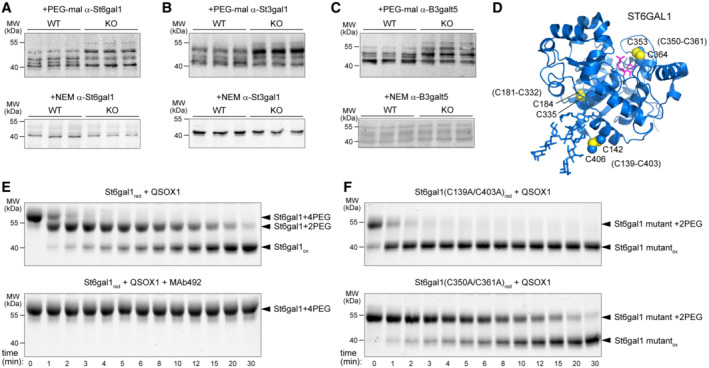
QSOX1 oxidizes Golgi glycosyltransferases Western blot analysis of colon epithelial cell lysates from WT and QSOX1 KO mice. Lysates were treated with PEG‐mal 2 kDa or NEM as indicated above each blot and probed using antibodies to St6gal1.As for panel (A) but using antibodies to St3gal1.As for panel (A) but using antibodies to B3galt5.Structure of the human ST6GAL1 catalytic domain in complex with CMP (PDB ID: 4JS2; Kuhn *et al*, 2013) with cysteine side chains shown as spheres and numbered. Numbering according to the murine St6gal1 sequence is indicated in parentheses.QSOX1 oxidizes St6gal1 *in vitro*. MAb492.1 is a monoclonal antibody that inhibits human QSOX1 (Grossman *et al*, 2013). St6gal1 with one pair of free cysteines is modified by two PEG‐mal additions (2PEG), whereas protein with two pairs of free cysteines acquires four PEG‐mal additions (4PEG). The change in migration per two PEG‐mal modifications appears greater than 4 kD as expected due to the differences in hydrodynamic properties and SDS binding of PEG vs. protein (Javitt *et al*, 2020b).QSOX1 oxidation of St6gal1 mutants. The C350‐C361 disulfide (the redox‐active disulfide present in the C139A‐C403A mutant) is oxidized rapidly, while the C142‐C406 disulfide (present in the C350A‐C361A mutant) is oxidized slowly. Data information: For the experiments in panels (A–C), equal amounts of total protein were applied to each gel lane, and no reducing agent was added. Experiments were repeated three times with fresh new lysates, and representative blots are shown. Source data are available online for this figure.

In addition to disulfide‐mediated crosslinking of mucins, another factor crucial to mucus function is glycosylation. Intriguingly, certain Golgi glycosyltransferases were reported to require disulfide bonds for function (Hirano *et al*, 2012; Hassinen *et al*, 2019; Ortiz‐Soto *et al*, 2019). We therefore examined the redox states of Golgi sialyltransferases in QSOX1 KO mice. Lysates from isolated colon epithelial cells were treated with polyethylene‐glycol modified maleimide (PEG‐mal) of 2 kDa to alkylate reactive cysteine thiol groups that were not protected in disulfide bonds. Proteins were then separated by SDS‐PAGE and western blotted for two sialyltransferases, St6gal1 and St3gal1, and for B3galt5, a galactosyltransferase that modifies core glycans (Zhou *et al*, 1999). In all three cases, species that migrated more slowly, indicating the presence of cysteines conjugated to PEG‐mal, were observed for QSOX1 KO samples (Fig 4A–C). It should be noted that PEG conjugation retards protein migration to a greater extent than addition of the equivalent mass of protein (Javitt *et al*, 2020b). As a control showing that the differences in glycosyltransferase migration between WT and KO are due to the addition of PEG‐mal, treatment with the small (125 Da) cysteine alkylating agent N‐ethylmaleimide (NEM) did not result in appreciable differences in migration (Fig 4A–C). Indicating that disulfide bonding defects are specific to certain glycosyltranferases, no differences were noted between WT and KO PEG‐mal‐treated lysates in the migration of a representative ER oxidoreductase (Pdia4) or a Golgi glycosyltransferase from the GALNT family (Galnt4; Appendix Fig S5). These proteins have at least as many luminal cysteines as the glycosyltransferases analyzed in Fig 4.

We next tested whether QSOX1 directly oxidizes Golgi sialyltransferases. Recombinant St6gal1 catalytic domain (Fig 4D) was prepared, reduced, and incubated with purified QSOX1 for various times before addition of PEG‐mal to quench oxidation reactions and label free thiols. St6gal1 was re‐oxidized by QSOX1 as measured by resistance to PEG‐mal modification (Fig 4E). Notably, only two of the three St6gal1 disulfides in the recombinant protein (C142–C406 and C353–C364) were reactive in this experiment because the third (C184–C335) performs a stabilizing role within the structure (Meng *et al*, 2013; Fig 4D and Appendix Fig S6) and is less accessible for reduction and modification. Of the two pairs of reduced cysteines, QSOX1 oxidized the pair adjacent to the St6gal1 active site much more rapidly than the pair involving the carboxy‐terminal cysteine (Fig 4E and F).

### Impaired glycosylation in colons of QSOX1 KO mice

**Figure 5:**
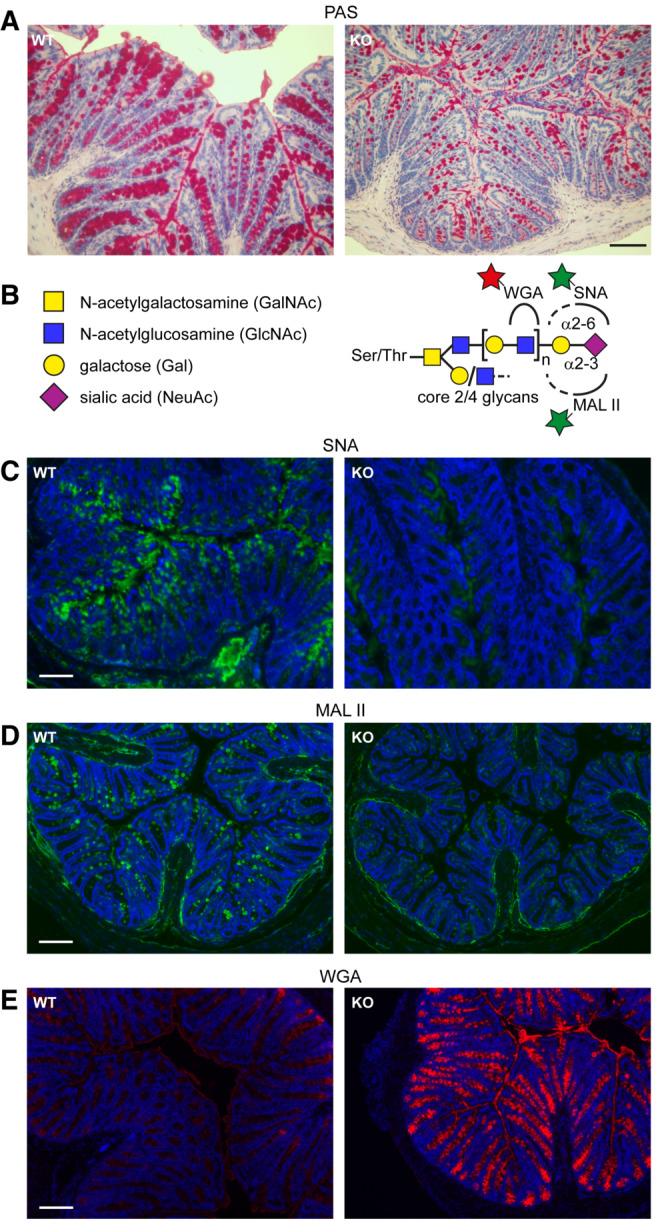
Altered glycan distribution in colons of QSOX1 KO mice Staining and labeling are representative images of three biological repeats.
PAS and hematoxylin staining of colon cross sections. Scale bar is 50 μm.Schematic showing the glycan species recognized by the indicated lectins. Stars represent fluorescent labels.Colon cross sections labeled with SNA lectin. Scale bar is 100 μm.Colon cross sections labeled with MAL II lectin. Scale bar is 100 μm.Colon cross sections labeled with WGA lectin. Scale bar is 100 μm.

The observed effect of QSOX1 on sialyltransferase redox state *in vivo* and *in vitro* would be functionally significant if manifested in altered glycosylation patterns. PAS staining is sensitive to glycan structures and was reported to decrease following sialidase treatment of colon sections (Roe *et al*, 1989). Though no major difference was detected in PAS in‐gel staining of reduced guanidine‐insoluble mucins (Fig 3B), PAS staining of colon sections was much stronger for WT than KO (Fig 5A). To further explore sialylation in QSOX1 KO colons, we used lectins that recognize relevant glycans (Fig 5B). *Sambucus nigra* agglutinin (SNA) binds sialic acid preferentially in a α‐2,6 linkage to terminal galactose (Shibuya *et al*, 1987), which is the product of St6gal1 activity. *Maackia amurensis* lectin II (MAL II) binds sialic acid in a α‐2,3 linkage (Wang & Cummings, 1988), the product of St3gal1 activity. Both SNA (Fig 5C and Appendix Fig S7A) and MAL II (Fig 5D) labeled WT colons much more strongly than KO colons, indicating perturbed sialylation in QSOX1 KO animals. The sialylation defects were specific, since an antibody recognizing sialyl‐Tn antigen (i.e., sialic acid on N‐acetylgalactosamine (GalNAc) O‐linked to serine or threonine), which is formed by the enzyme St6galnac1 (Marcos *et al*, 2011; Fig 5F), labeled WT and KO colon tissue similarly (Appendix Fig S7B and C).

**Figure 6:**
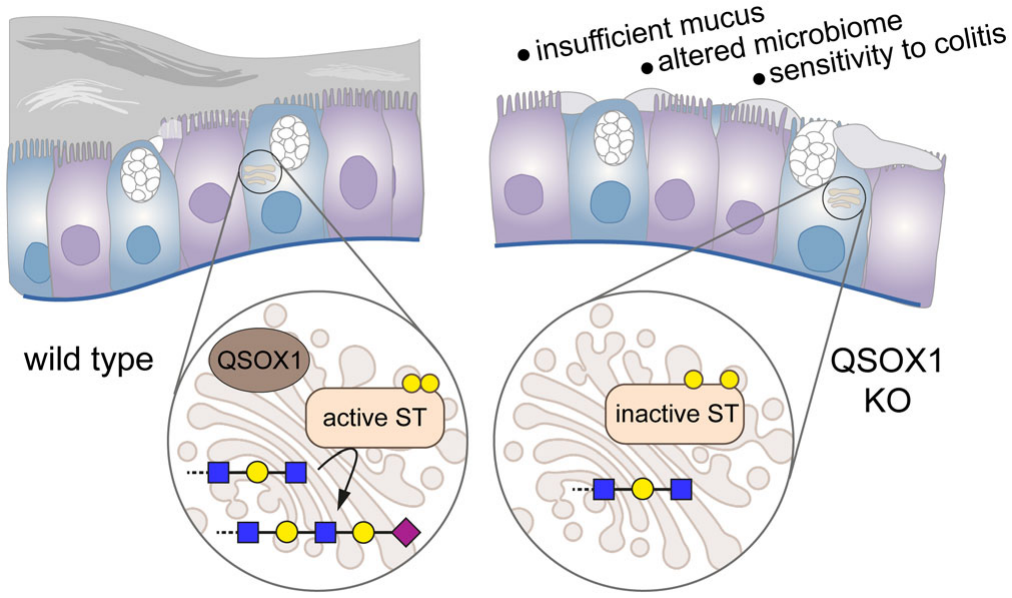
Role of Golgi QSOX1 and phenotypic effects of QSOX1 KO in the colon Schematic diagram showing the effect of QSOX1 KO on sialylation in the Golgi and the impact on colon function. ST stands for sialyltransferase.

Depressed sialylation would be expected to result in accumulation of precursor glycans. Specifically, GlcNAc would remain exposed without the coordinated action of galactosyl and sialyltransferases, which introduce the galactose and sialic acid terminal sugars (Fig 5B). Indeed, wheat germ agglutinin (WGA), which recognizes GlcNAc (Fig 5B), showed the opposite pattern to SNA and MAL II, labeling QSOX1 KO colons much more strongly than WT (Fig 5E and Appendix Fig 7D). Confirming that WGA labeling was due to GlcNAc binding rather than to other known activities of WGA (Ryva *et al*, 2019), a succinylated version of WGA that is more specific for GlcNAc (Monsigny *et al*, 1980) showed similarly enhanced labeling of QSOX1 KO colons (Appendix Fig S7E and D). Together, our data reveal that QSOX1 activates Golgi sialyltransferases through disulfide bond formation, promoting mucosal glycoprotein sialylation and stabilizing the mucus hydrogel (Fig 6).

## Discussion

The complex task of guarding the huge digestive and respiratory mucosal surfaces of the body requires a correspondingly complex macromolecular defense system. Thus, mucins have evolved diverse features to support their crucial protective functions. One is their ability to acquire intermolecular disulfide crosslinks and form polymers (Meldrum *et al*, 2018; Thornton *et al*, 2018). Another feature is modification with thousands of O‐linked glycans per mucin molecule (Arike & Hansson, 2016), facilitating mucus hydration and protecting the mucins against proteolytic degradation. Using these attributes, the multi‐faceted mucin molecules form the physical and functional scaffold of the mucosal barrier.

Mice lacking Muc2 (Van der Sluis *et al*, 2006), Agr2 (Park *et al*, 2009; Zhao *et al*, 2010) or having mutations in Muc2 that interfere with initial mucin folding and assembly (Heazlewood *et al*, 2008) develop spontaneous colitis or display rectal prolapse. QSOX1 KO mice did not display these symptoms, suggesting that early steps in mucin biosynthesis, which occur in the ER, progress normally in these animals. Based on our observation that colon mucus is compromised in QSOX1 KO mice, it was reasonable to speculate that disulfide‐mediated polymerization of mucins in the Golgi apparatus (Perez‐Vilar *et al*, 1998) would be defective. It was possible that impaired mucin polymerization led to the secretion of weak and poorly effective mucus, such that the QSOX1 KO mice had relatively normal gut function when unchallenged but were hypersensitive to induced colitis. Despite extensive investigation, however, we were not able to obtain evidence for a mucin disulfide‐mediated polymerization defect.

It was previously observed that undermining O‐glycosylation in the gut leads to colitis. Mice lacking core 1 glycans, or both core 1 and core 3 glycans, exhibited spontaneous disease (Fu *et al*, 2011; Bergstrom *et al*, 2017), whereas mice lacking only core 3 glycans were hypersensitive to induced colitis (An *et al*, 2007). In contrast to the poor staining of mucins extracted from the mice unable to produce core glycans (Bergstrom *et al*, 2017), PAS and Alcian blue staining of the guanidine‐insoluble fraction from QSOX1 KO colon extracts was indistinguishable from WT samples, indicating normal core glycan production in the QSOX1 KO mice (Fig 3B).

Building on the core O‐glycans, colon mucins become highly elaborated by the addition of glucose and galactose derivatives, and the glycan chain is finally capped by fucosylation and sialylation. Inspired by the observation that decreased disulfide bonding in certain sialyltransferases (STs) interferes with sialylation of N‐ and O‐linked glycans in cultured cell lines (Hassinen *et al*, 2019), we considered whether STs could be targets of QSOX1 activity in the colon. It is possible that the dependence of ST disulfides on oxygen tension (Hassinen *et al*, 2019) is due to the requirement for oxygen to drive disulfide formation by QSOX1 (Hoober *et al*, 1996). By analyzing protein redox states in colon epithelial cells, we found that cysteines of Golgi STs are indeed under‐oxidized in the absence of QSOX1 (Fig 4A–C). Moreover, we showed that the pathway linking disulfide formation to sialylation is critical for mucus stability and colon resistance to disease.

Sialyltransferases are a large enzyme family, with 20 members in humans and mice (Harduin‐Lepers *et al*, 2001; Lopez *et al*, 2017). The size of the family likely represents the need for tissue‐specific expression, temporal regulation, and specificity for distinct substrate sets. All ST family members retain a conserved pair of cysteines that participate in a structural disulfide bond (Rao *et al*, 2009). However, the amino acid sequences, including the presence and position of other cysteine residues, have diverged quite substantially (Appendix Fig S6). Consequently, we do not expect that all members of the ST family will respond uniformly to QSOX1 activity. This diversity of potential redox handles offers the possibility of independently regulating sialylation of different substrates. Moreover, it was reported that sialylation can be tuned by differential localization of various STs to particular Golgi or post‐Golgi compartments (Kitano *et al*, 2021). Interpreted together with the observations reported here, the types and extent of sialylation may be a complex outcome of the levels of STs, their inherent capacity for redox regulation, the physical and temporal extent of their co‐localization with QSOX1, and the level of QSOX1 activity in the cell.

The importance of proper sialylation for mucus barrier integrity observed in our studies is reinforced by the finding that human ST mutations correlate with inflammatory bowel disease (Yao *et al*, 2022). In a mouse model of a human mutation, deficiency in St6galnac1, a highly conserved ST that generates sialyl‐Tn antigen, resulted in phenotypes closely resembling those of QSOX1 KO animals: a compromised mucus barrier, massive decrease in colon protein sialylation, changes in microbiome composition, and susceptibility to colitis (Yao *et al*, 2022). In our experiments, antibody labeling of sialyl‐Tn showed comparable signal in WT and QSOX1 KO colons (Appendix Fig S7C and D), suggesting that St6galnac1 may not be a regulatory target of QSOX1. Nevertheless, the convergence of phenotypes in St6galnac1‐defective and QSOX1 KO mice demonstrate that sialylation of multiple types is critical for gut health, and that STs can be perturbed both by mutation and by dysregulation.

QSOX1‐mediated redox regulation of Golgi STs is expected to influence health and disease in contexts beyond the colon epithelium. For example, altered sialylation is a feature of cancer progression (Garnham *et al*, 2019; Buffone & Weaver, 2020). In many cases, ST expression levels increase in cancer (Pietrobono & Stecca, 2021), but it is possible that these enzymes are also activated post‐translationally, since increased QSOX1 expression is seen in many cancer types (Antwi *et al*, 2009; Soloviev *et al*, 2013; Knutsvik *et al*, 2016; Baek *et al*, 2018; Sung *et al*, 2018). Inhibition of extracellular QSOX1 using monoclonal antibody inhibitors (Grossman *et al*, 2013, 2016) was found to slow tumor growth and decrease metastasis (Feldman *et al*, 2020), apparently by modulating ECM properties rather than Golgi redox state. Importantly, we show here that administration of QSOX1 inhibitory antibodies did not potentiate DSS‐induced colitis (Fig 1B), demonstrating that QSOX1 activity in colon physiology, and probably the Golgi functions of QSOX1 in general, are protected from inhibition by systemic inhibitory antibody treatment. Potential use of QSOX1 inhibitory antibodies as cancer therapy would thus not be expected to produce colitis as a side effect. It remains to be determined whether the increase in sialylation in cancer is linked to increased Golgi QSOX1, and whether this pathway would also be a valuable target for inhibition.

The key finding of this work is that the Golgi‐localized and secreted enzyme QSOX1 contributes to intestinal physiology by regulation of Golgi glycosyltransferases rather than by direct introduction of disulfide bonds during glycoprotein polymerization as hypothesized by us and others (Mennerich *et al*, 2019; Dong & Springer, 2021). Our studies exposed an enzymatic, redox‐controlled pathway for sialylation in the Golgi, which, due to the widespread expression of QSOX1 (Tury *et al*, 2006) and STs (Kitagawa & Paulson, 1994; Harduin‐Lepers *et al*, 2001), is likely to play a role in other physiological processes in addition to the preservation of normal colon physiology and microbiome demonstrated here.

## Materials and Methods

### Reagents and Tools table

**Table:** 

Reagent or resource	Source	Identifier
**Antibodies, lectins, and stains**		
Rabbit anti mouse QSOX1	This study	N/A
Mouse anti GM130	BD Bioscience	Cat# 610823
Mouse anti mouse QSOX1 (316.1)	Grossman *et al* (2016)	N/A
Mouse anti human QSOX1 (492.1)	Grossman *et al* (2013)	N/A
Rabbit anti St6Gal1	Sigma Aldrich	Cat# SAB4502780
Rabbit anti St3Gal1	Aviva Systems Biology	Cat# ARP45410_P050
Rat anti B3Galt5	GeneTex	Cat# GTX48036
Mouse anti Sialyl Tn	Abcam	Cat# Ab115957
Rabbit anti VWF	Dako	Cat# A0082
Rabbit anti mouse Muc5b	Zhu *et al* (2008)	N/A
Rabbit anti ERp72	Abcam	Cat# Ab155800
Mouse anti C1Galt1	Santa Cruz	Cat# sc‐100745
Rabbit anti Galnt4	Proteintech	Cat# 12897‐1‐AP
Rabbit anti MUC2 C‐term	GeneTex	Cat# GTX100664
Rabbit anti human MUC2‐D1	This study	N/A
Rabbit anti mouse Muc2‐D3	Javitt *et al* (2020a)	N/A
Goat anti mouse‐Alexa Fluor 488	Abcam	Cat# Ab150113
Goat anti mouse‐Alexa Fluor 568	Abcam	Cat# Ab175473
Goat anti rabbit‐Alexa Fluor 488	Abcam	Cat# Ab150077
Goat anti rabbit‐Alexa Fluor 568	Abcam	Cat# Ab175471
Sambucus nigra lectin (SNA)‐fluorescein	Vector Laboratories	Cat# Fl‐1301‐2
Maackia amurensis lectin II (MAL II)‐biotinylated	Vector Laboratories	Cat# B‐1265
Wheat germ agglutinin‐Alexa Fluor 488	ThermoFischer	Cat# W6748
Succinylated wheat germ agglutinin‐biotinylated	Vector Laboratories	Cat# B‐1025S‐5
Wheat germ agglutinin‐biotinylated	Vector Laboratories	Cat# B‐1025‐5
DAPI	Merck	Cat# 10236276001
Goat anti rabbit‐HRP	Jackson ImmunoResearch	Cat# 111‐035‐003
Goat anti mouse‐HRP	Jackson ImmunoResearch	Cat# 115‐035‐062
Goat anti rat‐HRP	Jackson ImmunoResearch	Cat# 112‐035‐062
Streptavidin‐Alexa Fluor 488	Jackson ImmunoResearch	Cat# 016‐540‐084
**Cell lines**		
MDA‐MB‐231	Feldman *et al* (2020)	N/A
FreeStyle™ 293‐F	Thermo Fisher Scientific	Cat# R79007
**Chemicals, Peptides, and Recombinant Proteins**		
Dextran sodium sulfate	TdB Consultancy	CAS# 9011‐18‐1
Alexa Fluor 488 labelled EUB	Sigma Aldrich	N/A
Liberase™	Sigma Aldrich	Cat# 5401119001
Periodic acid Schiff (PAS) kit	Sigma Aldrich	Cat# 395B
Alcian blue 8GX	Sigma Aldrich	Cat# A3157‐10g
jetPRIME	Polysciences, Inc.	Cat# 114‐15
N‐Ethylmaleimide	Sigma Aldrich	Cat# E3876
PEG‐mal 2 kDa	Iris‐Biotech	Cat# PEG1147.0001
**Embryonic stem cells and mice**		
QSOX1 specific embryonic stem cells	KOMP Repository	QSOX1^tm1a(KOMP)Wtsi^
C57Bl mice	Bred in house	N/A
QSOX1 KO mice	Bred in house	N/A
**Recombinant DNA**		
MUC2 D1D2D3CysD1 expression plasmid	Javitt *et al* (2019)	Addgene ID: 155214
MUC2 D1D2D3CysD1 C1088A expression plasmid	This study	N/A
MUC2 D1D2D3CysD1 C1030A expression plasmid	This study	N/A
MUC2 D1D2D3CysD1 C1088A/C1130A expression plasmid	This study	N/A
QSOX1 expression plasmid	Horowitz *et al* (2018)	N/A
St6gal1 expression plasmid	This study	N/A
St6gal1 C139A/C403A expression plasmid	This study	N/A
St6gal1 C350A/C361A expression plasmid	This study	N/A
**Cell culture reagents**		
PBS	Sigma Aldrich	Cat# D6882
RPMI‐1640	Sigma Aldrich	Cat# R8758
DMEM	Sigma Aldrich	Cat# D6429
FreeStyle™ 293 expression medium	ThermoFisher	Cat# 12338002
Pen‐Strep solution	Biological Industries	Cat# 03‐031‐1B
L‐glutamine solution	Biological Industries	Cat# 03‐020‐1B
Foetal Bovine Serum	Biological Industries	Cat# 04‐007‐1A
**Other**		
PowerSoil DNA isolation kit	MO BIO Laboratories	Cat# 12888‐50
Cell strainer	SPL life sciences	Cat# 93070
GeneChip mouse gene 2.0 ST array	ThermoFisher	Cat# 902118
RNeasy mini kit	Qiagen	Cat# 74104
Chromium Single Cell 3′ GEM Kit	10× Genomics	Cat# PN‐1000092

### Methods and Protocols

#### Mice

QSOX1 knockout (KO) mice were generated from the embryonic stem cell clone QSOX1^tm1a(KOMP)Wtsi^ obtained from KOMP Repository. The background strain is C57BL/6. QSOX1 deletion was validated by mRNA and protein detection, and all mice were genotyped. WT and QSOX1 KO pairs in each of the experiments described in this work were littermates. Aside from the microbiome experiment, littermates were not necessarily co‐housed. All experiments were approved by the institutional animal care and use committee (IACUC) of the Weizmann Institute of Science. Approval numbers: 05480818‐2, 01280121‐2. Mice were housed in the Weizmann Institute mice facility, no more than five mice per cage, with the exception of the microbiome study, with access to food and water *ad libitum*, and wellbeing was checked daily. For all experiments, unless otherwise specified, colons were collected from 3‐month‐old QSOX1 KO mice and WT littermates.

#### Cell lines

MDA‐MB‐231 breast cancer cells were obtained from Prof. Yosef Yarden, Weizmann Institute of Science. Cells were maintained in DMEM (Sigma Aldrich) supplemented with 10% fetal bovine serum, L‐glutamine, and Pen‐Strep (all from Biological Industries). Cells were tested for mycoplasma contamination but were not authenticated.

#### Colon epithelial cell isolation

The isolation protocol for colon epithelial cells was adopted from a published protocol (Bahar Halpern *et al*, 2020). Colons were removed, opened longitudinally, and incubated for 10 min in 5 ml of 10 mM ethylenediaminetetracetic acid (EDTA) in phosphate buffered saline (PBS) on ice. Colons were then moved to 5 ml of 100 μg/ml Liberase (Sigma) in PBS and incubated for 2–3 h at 37°C with shaking. Finally, suspended material was passed through a 100 μm cell strainer, and cells were washed in PBS. For immunofluorescence and lectin labeling, cells were resuspended in RPMI‐1640 medium supplemented with serum and antibiotics, then allowed to recover overnight (ON) at 37°C with 5% CO_2_.

#### Immunofluorescence and lectin labeling of cells

For immunofluorescence labeling, cells were transferred to glass cover slips in 24‐well plates and after 1 h at 37°C were fixed for 20 min at room temperature (RT) with 3.7% formaldehyde. Following fixation, cells were permeabilized with 0.1% Triton X‐100 in PBS for 2 min, washed, and incubated for 1 h with 5% BSA in PBS containing 0.1% Tween (PBST). Cells were then incubated with primary antibody for 1 h at RT, washed three times with PBST, and incubated with fluorescently‐labeled secondary antibody and DAPI for 1 h at RT. Following additional three washes, cover slips were placed, cells face down, onto a 5 μl drop of ProLong Gold antifade reagent (Invitrogen) on glass slides and were left to dry ON. For fluorescent lectin labeling (SNA‐fluorescein and WGA‐Alexa488), cells were fixed with formaldehyde, washed three times, and blocked with 3% BSA in PBS. Cells were then incubated with lectin (1:200 in PBST) and DAPI for 1–2 h, washed three times, covered by mounting media, sealed with a cover slip, and allowed to dry ON prior to imaging. Biotinylated lectin labeling (WGA‐biotin, succinylated WGA‐biotin, and MAL II‐biotin) was done similarly except an additional incubation with streptavidin‐Alexa488, followed by washing, was performed.

#### Light microscopy

All fluorescent light microscopy imaging, except for longitudinal sections, was done using an Olympus IX51 microscope equipped with Olympus XM10 camera. Images were processed using ImageJ software.

#### Dextran sulfate sodium (DSS)‐induced acute colitis

Acute colitis was induced by administration of DSS (2.5% in drinking water) for 5 days to 4 WT and 4 QSOX1 KO, 8‐week‐old female mice. Disease severity was assessed and scored on days 2, 4, and 5, as follows: 1 = normal stool consistency, no rectal bleeding, 2 = loose stool, no rectal bleeding, 3 = diarrhea, minor rectal bleeding, and 4 = gross rectal bleeding and prolapse. Mice were sacrificed at the experiment endpoint (day 5), colons were removed from cecum to rectum, and their lengths were measured. Distal colon was then separated and fixed for 2 h in fresh, cold Carnoy's solution (60% ethanol, 30% chloroform, 10% acetic acid) at 4°C (Swidsinski *et al*, 2005). Following fixation, colons were washed in 100% ethanol and embedded in paraffin. Cross sections of distal colon were H&E stained, imaged, and analyzed. To test the potentiation of DSS‐induced colitis by systemic QSOX1 inhibition, 30 mg/kg MAb316.1, an inhibitory monoclonal antibody for murine QSOX1 (Grossman *et al*, 2016), was administered twice a week, for 3 weeks, via intraperitoneal injection to 5 WT 6‐week‐old female mice. A control group received control IgG in the same regimen. DSS was added to drinking water during the third week of antibody injection, and colitis was evaluated as described above.

#### Microbiome analysis

Two cages of 6‐week‐old female mice were established, each containing 3 WT and 3 QSOX1 KO mice. Colons were removed at 8 weeks of age, and feces (~200 mg) closest to the rectum were collected into 200 μl lysis buffer (150 mM NaCl, 0.5% sodium deoxycholate, 0.1% Triton X‐100, 50 mM Tris–HCl, pH 8.0, and protease inhibitors). Samples were lysed using Tissuelyzer LT bead mill (Qiagen), and DNA was purified using the MO BIO PowerSoil kit according to manufacturer's instructions. Twenty microliter of 50 ng/μl DNA of each sample were further processed for microbiome analysis by MR DNA (Molecular Research LP, Shallowater, TX, USA). Primers for the 16S rRNA gene V4 variable region 515/806 were used for amplification. Sequencing was performed on an Ion Torrent PGM following manufacturer's guidelines. Sequence data were processed using a proprietary analysis pipeline. Barcodes, primers, and sequences less than 150 bp were removed. Sequences with ambiguous base calls and with homopolymer runs exceeding 6 bp were also removed. Sequences were denoised, operational taxonomic units (OTUs) were generated, and chimeras were removed. OTUs were defined by clustering at 3% divergence (97% similarity). Final OTUs were taxonomically classified using BLASTn against a database derived from RDPII and NCBI. Microbiome data were evaluated in a multivariate manner to determine the changes between the two groups. Alpha and beta diversity of the bacterial community structure was calculated, and specific bacterial Genera differences between groups was evaluated using controlled ANOVA.

#### Transcriptome microarray and single‐cell expression analyses

For microarray analysis, colons from 3 WT and 3 QSOX1 KO female mice were removed, and total RNA was immediately extracted using the RNeasy mini kit (Qiagen). RNA quality was assessed using the Bioanalyzer 2100 platform (Agilent), and samples were then processed and hybridized to GeneChip mouse gene 2.0 ST array according to the manufacturer's instructions. For single‐cell expression analysis, colon epithelial cells were isolated as described above, and 20,000 cells were analyzed using the 10× Genomics Chromium Single Cell Gene Expression platform according to the manufacturer's instructions. Marker genes were found by differential expression based on the non‐parametric Wilcoxon rank sum test (Seurat default). Clustering was done using KNN graph, and non‐linear reduction was done using UMAP.

#### Colon section preparation and labeling

With the exception of SNA labeling, immunofluorescence and lectin labeling of colons was done on paraffin sections. Colons were removed and immediately placed in fresh, cold Carnoy's fixative solution for 2 h at 4°C, washed in 100% ethanol, and embedded in paraffin. For immunofluorescence labeling, sections were deparaffinized, and antigen retrieval was performed by incubation in 10 mM sodium citrate, pH 6, for 20 min at 95°C. Slides were then washed in PBS, blocked in 3% BSA in PBS, and incubated with primary antibody (1:50–1:100 in PBST). Slides were washed again in PBS, incubated with fluorescently labeled secondary antibody (1:200 in PBST) and DAPI, and washed again. For both immunofluorescence and lectin labeling, sections were covered by mounting media, sealed with a cover slip, and allowed to dry ON prior to imaging. For WGA labeling, deparaffinized sections were blocked in 3% BSA in PBS and incubated with WGA‐Alexa Fluor 488 (1:100 in PBST) and DAPI. For MAL II labeling, deparaffinized sections were blocked in 3% BSA in PBS and incubated with MAL II‐biotin (1:100 in PBST). Slides were then washed and incubated with streptavidin‐Alexa Fluor 488 (1:200 in PBST) and DAPI. For S16 probe labeling, colon sections were deparaffinized, washed in PBS, and incubated with 100 μl of fluorescently labeled EUB (A488‐GCTGCCTCCCGTAGGAGT) ON at 4°C protected from light. Slides were then washed in PBST, incubated with DAPI for 1 h, washed, covered by mounting media, and sealed with a cover slip.

For SNA staining in frozen sections, colons were removed, dissected into 2 mm sections, placed in cold tissue mold, covered with optimal cutting temperature (OCT) compound, immediately placed in liquid nitrogen, and then stored at −80°C. For sectioning, frozen blocks were placed in a cryotome cryostat, and 10 μm thick sections were cut, placed on glass slides, and stored at −80°C until labeling. Sections were fixed with 3.7% formaldehyde for 30 min at RT, washed three times in PBS, and incubated for 1 h in 3% BSA in PBS. Sections were then incubated SNA‐fluorescein (1:200 in PBST) and DAPI for 1 h at RT, followed by washing three times with PBST. Five microliter of ProLong Gold antifade reagent (Invitrogen) was spread on a coverslip, which was then placed on the tissue section and left to dry ON prior to imaging.

#### Longitudinal sectioning of colon and immunofluorescence labeling

Freshly removed colons were cut open longitudinally, gently washed with PBS containing Mg^2+^ and Ca^2+^, and covered by fresh, cold Carnoy's fixative solution for 2 h at 4°C. Colons were then washed with PBS, incubated with primary antibody for 1 h (1:50) in 0.1% PBST, washed again, and incubated with fluorescently labeled secondary antibody (1:200 in PBST) and DAPI. Following additional washing, the colons were covered by mounting media, sealed with a cover slip, and allowed to dry ON prior to imaging. Confocal imaging was performed using an upright Leica TCS SP8 (Leica microsystems CMS GmbH, Germany) at the Advanced Optical Imaging Unit, de Picciotto‐Lesser Cell Observatory Unit of the Moross Integrated Cancer Center Life Science Core Facilities, Weizmann Institute of Science.

#### Transmission electron microscopy

For preparation of thin cell sections, mouse colons were fixed using 2.5% gluteraldehyde, 2% paraformaldehyde in 0.1 M sodium cacodylate buffer, pH 7.4, at RT, washed in cacodylate buffer at 4°C, and post‐fixed with 1% osmium tetroxide in cacodylate buffer for 1 h, then incubated with 2% uranyl acetate for 1 h. Samples were dehydrated in cold ethanol and then embedded in Epon. Thin sections were cut and stained with 2% uranyl acetate and Reynold's lead citrate. Samples were visualized using a Tecnai T12 electron microscope (Thermo Fisher Scientific) equipped with a OneView camera (Gatan).

#### Scanning electron microscopy

Freshly removed colons were cut longitudinally, gently washed with PBS, and covered by fresh, cold Carnoy's fixative solution for 1 h at 4°C. Following fixation, samples were gently rinsed in PBS and incubated in increasing concentrations of ethanol (30–100%). Samples in absolute ethanol were dried using critical point dehydration and stored under vacuum ON. Finally, samples were coated with 4 nm iridium and imaged using an ULTRA 55 FEG microscope (Zeiss).

#### Insoluble mucin staining

Colons were removed, opened longitudinally, dissected into small pieces, and immediately placed in 0.5 ml cold lysis buffer containing 3 M guanidinium chloride. Colons were homogenized in a Tissuelyzer LT bead mill, and tissue debris was removed by 3 min centrifugation at 100 *g*. The resulting supernatants were centrifuged for 10 min at 17,000 *g*, and the pellet was resuspended in 8 M urea dissolved in 20 mM Tris, pH 7.4, containing 50 mM tris(hydroxymethyl)phosphine (THP) and incubated at RT for 1 h without agitation. The tube was inverted gently twice during this incubation. Remaining insoluble material was removed by centrifugation for 2 min at 17,000 *g*, and NEM was added to the supernatant to a concentration of 30 mM. After 1 h at RT, aliquots were taken for separation on 1.5 mm thick 6% SDS‐PAGE gels. After electrophoresis, gels were stained with Alcian blue and with PAS (Møller & Poulssen, 2002; Packer *et al*, 2002).

#### Periodic Acid‐Schiff (PAS) staining of colon sections

Paraffin colon sections were deparaffinized and immersed in Periodic acid solution for 5 min at RT followed by three washes with water. Slides were then immersed in Schiff's reagent for 15 min at RT followed by washing in running tap water for 5 min. Finally, slides were counterstained in hematoxylin solution for 1 min, washed with running tap water, covered by mounting media, sealed with a cover slip, and allowed to dry ON prior to imaging.

#### Western blots of Muc5b and Vwf polymers

For Muc5b analysis, lung lavage in saline was performed on WT and QSOX1 KO mice. Dot blots were performed to assess Muc5b levels in each lavage, and samples containing approximately equivalent amounts of Muc5b were separated on an SDS/agarose gel. After transfer of proteins from the gel to PVDF membrane by vacuum blot, the blot was probed with rabbit‐anti‐mouse Muc5b antibody (Zhu *et al*, 2008; Roy *et al*, 2014).

For Vwf analysis, fresh blood samples were immediately mixed 1:10 with 3.2% sodium citrate pH 7.4, and plasma was collected after a 4,000 *g* spin for 20 min at RT. Plasma samples were diluted 1:20 and run on an SDS/agarose gel. After transfer of proteins from the gel to PVDF membrane by vacuum blot, the blot was probed with a rabbit‐anti‐VWF antibody.

#### Analysis of MUC2 fragment dimerization

MDA‐MB‐231 cells were grown in 24‐well plates. QSOX1‐specific or scrambled siRNA oligonucleotides (20 nM) were transfected into the cells using jetPRIME reagent (Polyplus‐transfection) according to the manufacturer's instructions. Cells were allowed to recover for 1 day before plasmid DNA (0.5 μg in 500 μl) encoding the N‐terminal segment of human MUC2 (Javitt *et al*, 2019) or mutants thereof was transfected into the same cells. Two days after siRNA transfection, culture medium was taken from transfected cell wells and analyzed by western blot. MUC2 was detected by polyclonal antibody raised against the MUC2 D3 region (Javitt *et al*, 2020a). Validation of QSOX1 knockdown in this experiment was done by immunofluorescence using a polyclonal antibody against human QSOX1 (Ilani *et al*, 2013).

#### Colon epithelial cell lysis

Freshly isolated colon epithelial cells were pelleted and lysed by adding 10% trichloroacetic acid to the cell pellet and incubating on ice for 20 min. Protein pellet was washed twice with cold acetone and dissolved in lysis buffer containing 10 mM of either PEG‐mal 2 kDa or NEM. Protein concentration in lysates was determined using bicinchoninic acid, and samples containing 30 μg of total protein were separated on SDS‐PAGE gels and western blotted for the indicated proteins.

#### St6gal1 preparation and oxidation assays

DNA coding sequence for amino acids 85 to 403 of murine St6gal1 was inserted downstream of the QSOX1 signal sequence (MRRCNSGSGPPPSLLLLLLWLLAVPGANA/AP, cleavage occurs at the slash), a His_6_ tag, and a TEV protease cleavage site in the pcDNA3.1 vector. Cysteine mutations were made in this plasmid using mutagenic PCR. The resulting plasmids were transiently transfected into HEK293F cells (ThermoFisher). Cells were maintained in FreeStyle 293 medium and transfected using the PEI Max reagent (Polysciences Inc.) with a 1:3 ratio (w/w) of DNA to PEI at a concentration of 1 million cells per ml. Six days after transfection, cells were removed from the cultures by centrifugation for 10 min at 500 *g*. The culture media were then further clarified by centrifugation for 15 min at 9,500 *g* and filtration through a 0.45‐μm pore‐size membrane. St6ga11 and mutants were purified from the filtered medium by nickel‐nitrilotriacetic acid (Ni‐NTA) chromatography and concentrated to about 10 μM.

To prepare reduced St6gal1 and mutants, the proteins were incubated with 20 mM THP for 20 min at RT. Reduced St6gal1 and mutants were then applied to a PD‐10 desalting column equilibrated with PBS plus 1 mM EDTA. The concentrations of the peak fractions were measured, and 200 μl reactions were set up containing 7 μM (WT) or 3.2 μM (mutants) reduced St6gal1 and initiated by addition of QSOX1 to a concentration of 100 nM. Aliquots (10 μl for WT and 12 μl for mutants) were removed from the reaction at the indicated times and added to 1 μl of 10 mM PEG‐mal 2 kDa. Gel loading buffer was added, and aliquots were applied to SDS‐PAGE prepared with 2,2,2‐trichloroethanol. After electrophoretic separation, protein bands were visualized according to a stain‐free protocol (Ladner *et al*, 2004).

## Author contributions

**Tal Ilani**: Conceptualization; investigation; visualization; methodology; funding acquisition; writing – review and editing. **Nava Reznik**: Investigation; writing – review and editing. **Noa Yeshaya**: Investigation; writing – review and editing. **Tal Feldman**: Investigation. **Patrick Vilela**: Investigation. **Zipora Lansky**: Investigation. **Gabriel Javitt**: Investigation. **Michal Shemesh**: Methodology; investigation. **Ori Brenner**: Methodology; validation. **Yoav Elkis**: Conceptualization; methodology. **Neta Varsano**: Methodology; investigation. **Ana M. Jaramillo**: Investigation. **Christopher M. Evans**: Supervision; writing – review and editing; funding acquisition. **Deborah Fass**: Conceptualization; supervision; investigation; funding acquisition; project administration; writing – original draft; writing – review and editing.

## Disclosure and competing interests statement

DF is an editorial advisory board member at The EMBO Journal. This has no bearing on the editorial consideration of this article for publication.

## Supporting information



Appendix

Source Data for Figure 3

Source Data for Figure 4

## Data Availability

RNA‐seq data have been deposited in the ArrayExpress database at EMBL‐EBI (www.ebi.ac.uk/arrayexpress) under accession number E‐MTAB‐11110.
