# Supplementary material for: The disulfide catalyst QSOX1 maintains the colon mucosal barrier by regulating Golgi glycosyltransferases
Source: EMBO J. 2022 Oct 17;42(2):e111869. doi: 10.15252/embj.2022111869 (PMC9841341; doi:10.15252/embj.2022111869)
Supplement: Supplementary file 1 — Appendix [file EMBJ-42-e111869-s004.pdf]

Appendix

The QSOX1 Disulfide Catalyst Maintains the Colon Mucosal Barrier by Regulating Golgi Glycosyltransferases

Table of contents

	Page
**Appendix Figure S1.** Single-cell analysis of QSOX1 expression in the colon.	2
**Appendix Figure S2.** Microbial ecology of WT and QSOX1 KO mice.	3
**Appendix Figure S3.** Summary of colon transcriptome data for WT and QSOX1 KO mice.	4
**Appendix Figure S4.** Additional images of colon longitudinal sections labeled for Muc2.	5
**Appendix Figure S5.** Control western blot analysis of colon epithelial cell lysates from WT and QSOX1 KO mice.	5
**Appendix Figure S6.** Sequence alignment of the catalytic domains of human sialyltransferases.	6
**Appendix Figure S7.** Additional lectin and antibody labeling of WT and QSOX1 KO colons and isolated epithelial cells.	7

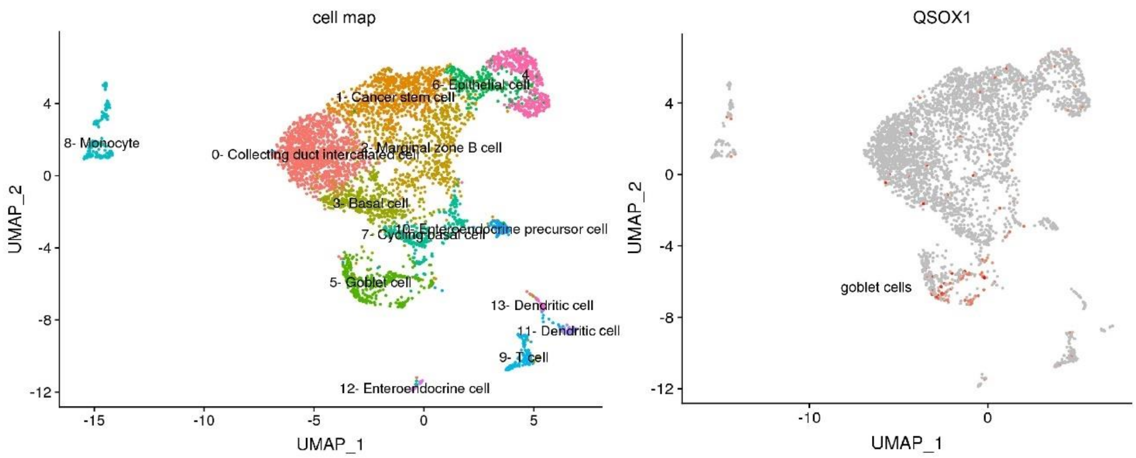

Appendix Figure S1. Single-cell analysis of QSOX1 expression in the colon.

Left, a reference uniform manifold approximation and projection (UMAP) plot shows cell-type clusters based on the expression of known marker genes. Right, UMAP plot of QSOX1 expression (red) in WT murine colon.

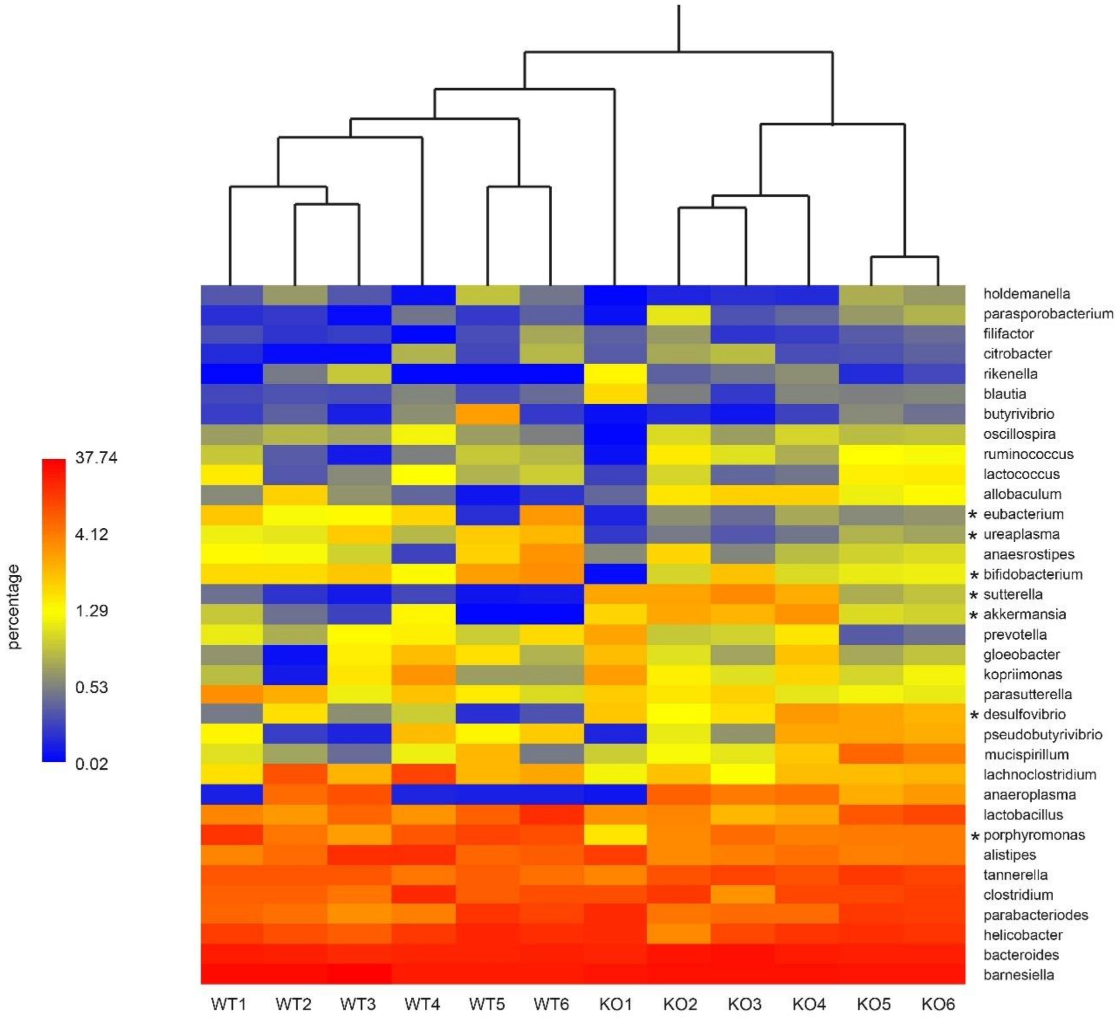

Appendix Figure S2. Microbial ecology of WT and QSOX1 KO mice.

The predominant genera detected in the WT and QSOX1 KO fecal samples are displayed and clustered in a hierarchal dendrogram. The percentages of the genera are colored according to the scale on the left. The genera indicated by asterisks are plotted in Figure 1F. At the top of the heat map, the lengths of the connecting lines are related to the similarities between the microbial consortia of each sample. WT and KO 1-3 were co-housed, and WT and KO 4-6 were co-housed prior to microbiome analysis. KO1 clusters loosely with the WT animals, but all other samples cluster according to genotype.

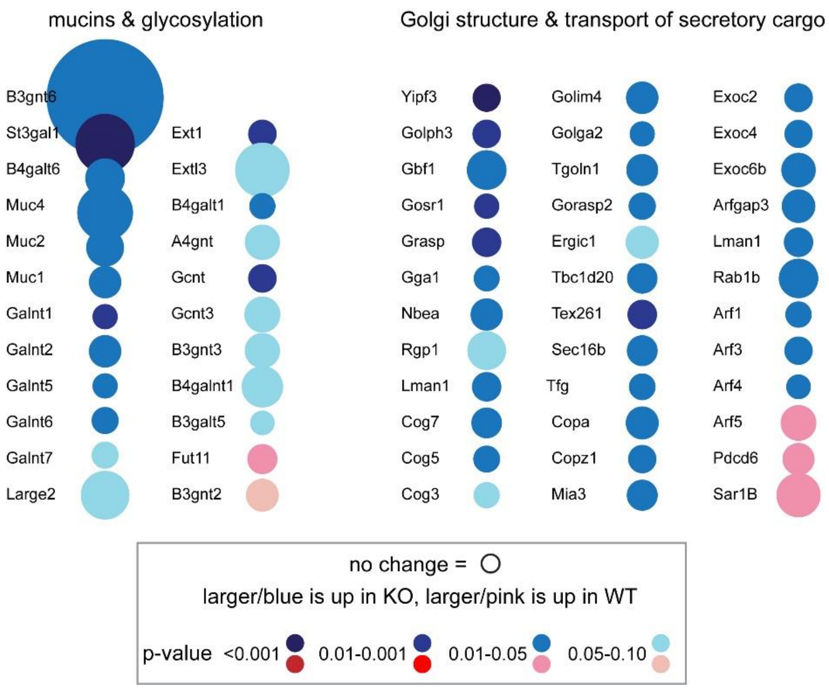

Appendix Figure S3. Summary of colon transcriptome data for WT and QSOX1 KO mice.

Changes in transcript levels of mucin genes and genes involved in glycosylation and vesicle transport are displayed. Circle diameter is proportional to fold change, blue/pink is up/down-regulation in the QSOX1 KO relative to WT. Darker shades indicate lower p-values. Data are from 3 WT and 3 QSOX KO mice.

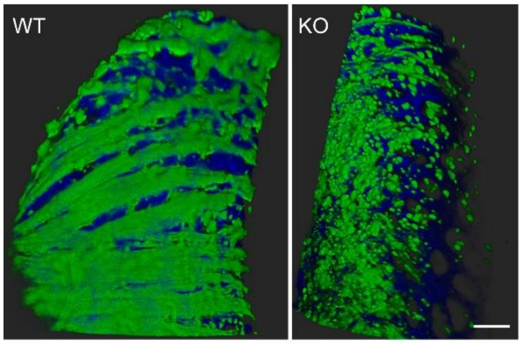

Appendix Figure S4. Additional images of colon longitudinal sections labeled for Muc2.

Even when regions of the WT and QSOX1 KO samples that have greater mucin coverage are selected, Muc2 in the KO does not appear as organized bundles. Scale bar is 50 µm.

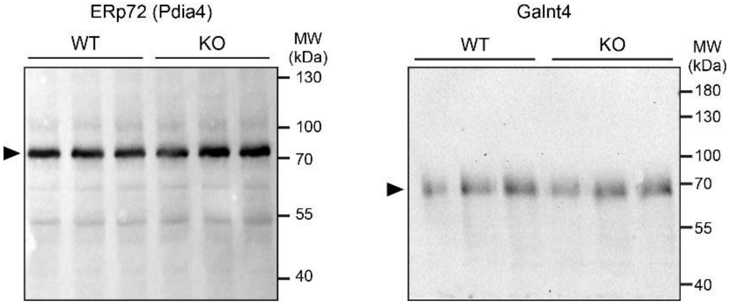

Appendix Figure S5. Control western blot analysis of colon epithelial cell lysates from WT and QSOX1 KO mice.

Lysates were treated with PEG-mal 2 kDa and probed using antibodies to ERp72 or Galnt4. No differences were seen in the migration of these proteins from QSOX1 KO mice compared to WT. The experiments were repeated twice with fresh new lysates, and representative blots are shown. Arrowheads indicate the bands at the expected size for each protein.

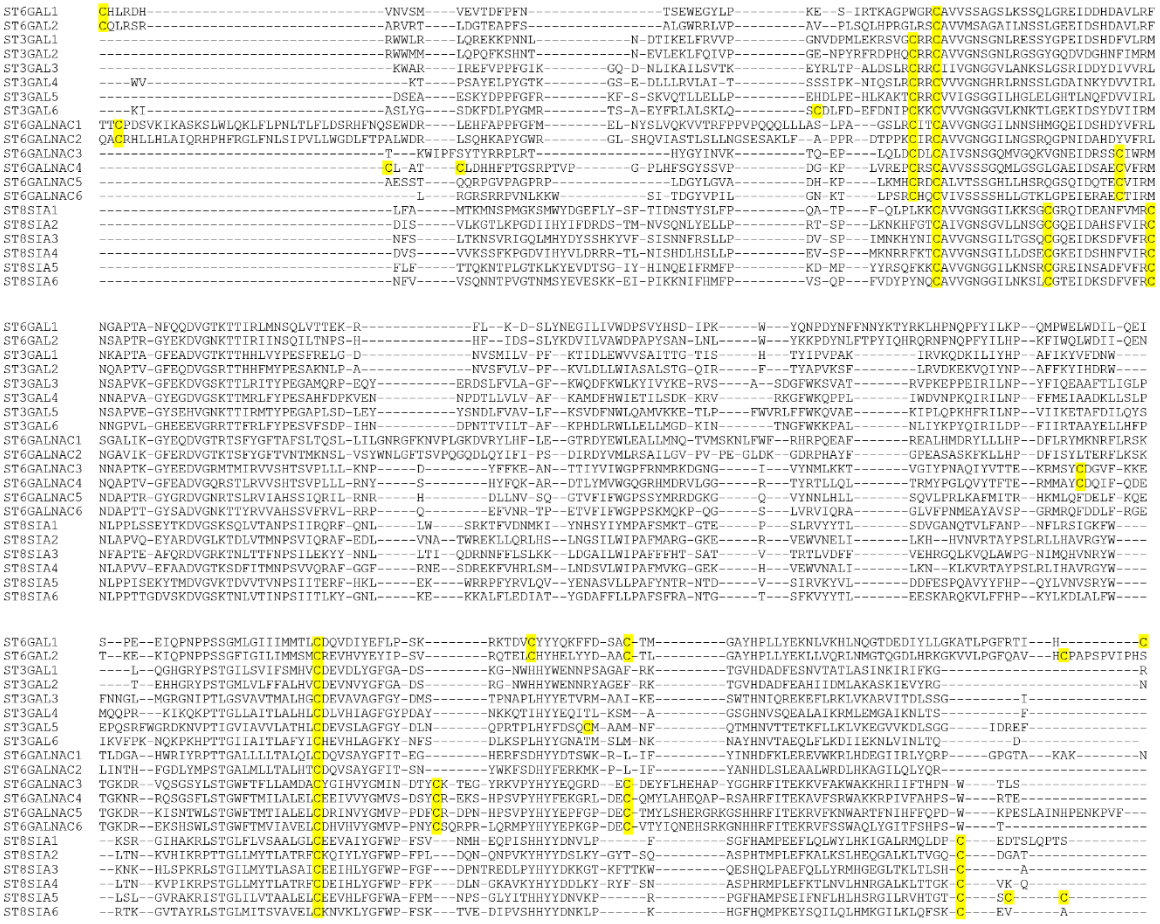

Appendix Figure S6. Sequence alignment of the catalytic domains of human sialyltransferases.

Cysteine amino acids are highlighted. Two cysteines forming a structural disulfide are conserved in all sequences.

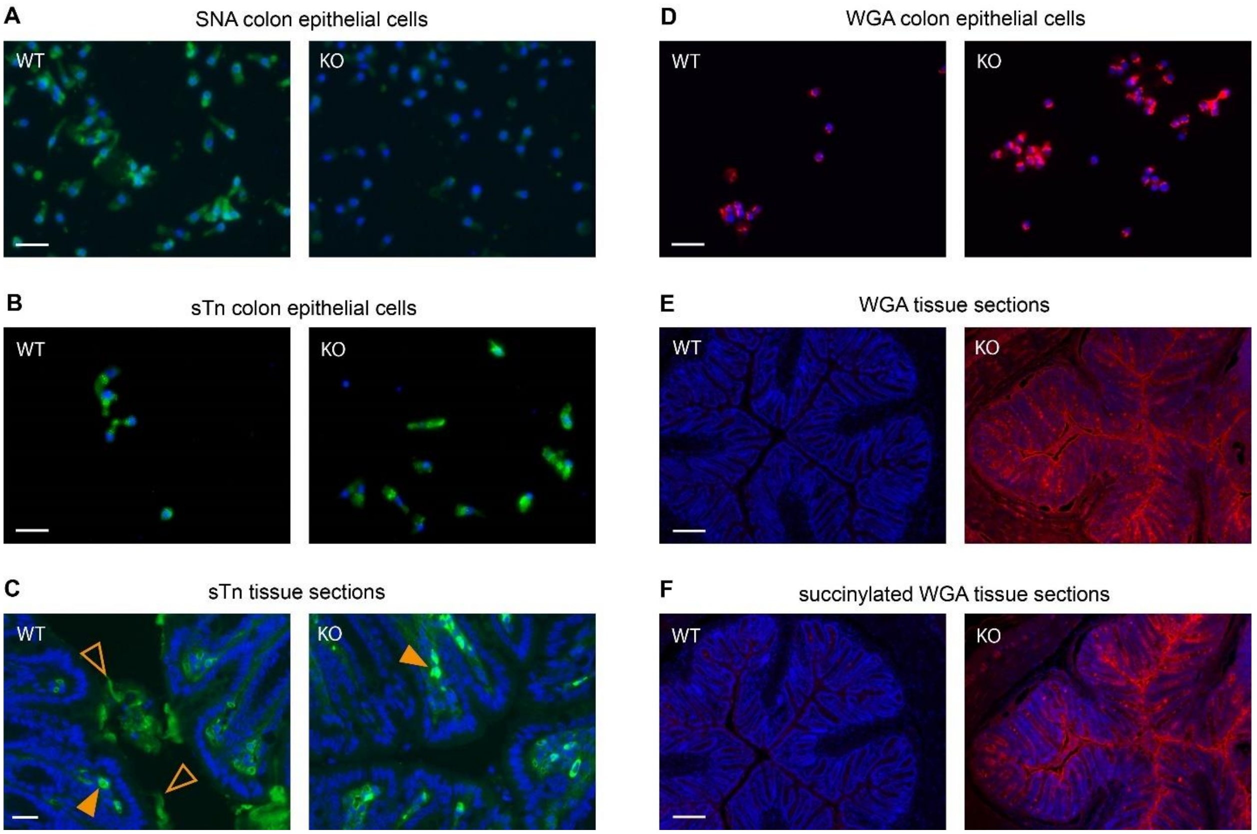

Appendix Figure S7. Additional lectin and antibody labeling of WT and QSOX1 KO colons and isolated epithelial cells.

A Isolated colon epithelial cells labeled with SNA. Scale bar is 20 µm.

B Isolated colon epithelial cells labeled with antibody recognizing α-sialyl Tn antigen (sTn). Scale bar is 20 µm.

C Colon cross sections labeled with sTn antibody. Labeling was seen within the tissue in both WT and KO colons (filled arrowheads), but luminal staining was seen only in WT colons (open arrowheads) because little mucus was present in QSOX1 KO colon lumen. Scale bar is 20 µm.

D Isolated colon epithelial cells labeled with WGA. Scale bar is 20 µm.

E,F Succinylated and non-succinylated biotinylated WGA both labeled KO colon sections much more strongly than WT colon sections. Scale bar is 100 µm.
