# Supplementary figures and images for: The disulfide catalyst QSOX1 maintains the colon mucosal barrier by regulating Golgi glycosyltransferases
Source: EMBO J. 2022 Oct 17;42(2):e111869. doi: 10.15252/embj.2022111869 (PMC9841341; doi:10.15252/embj.2022111869)

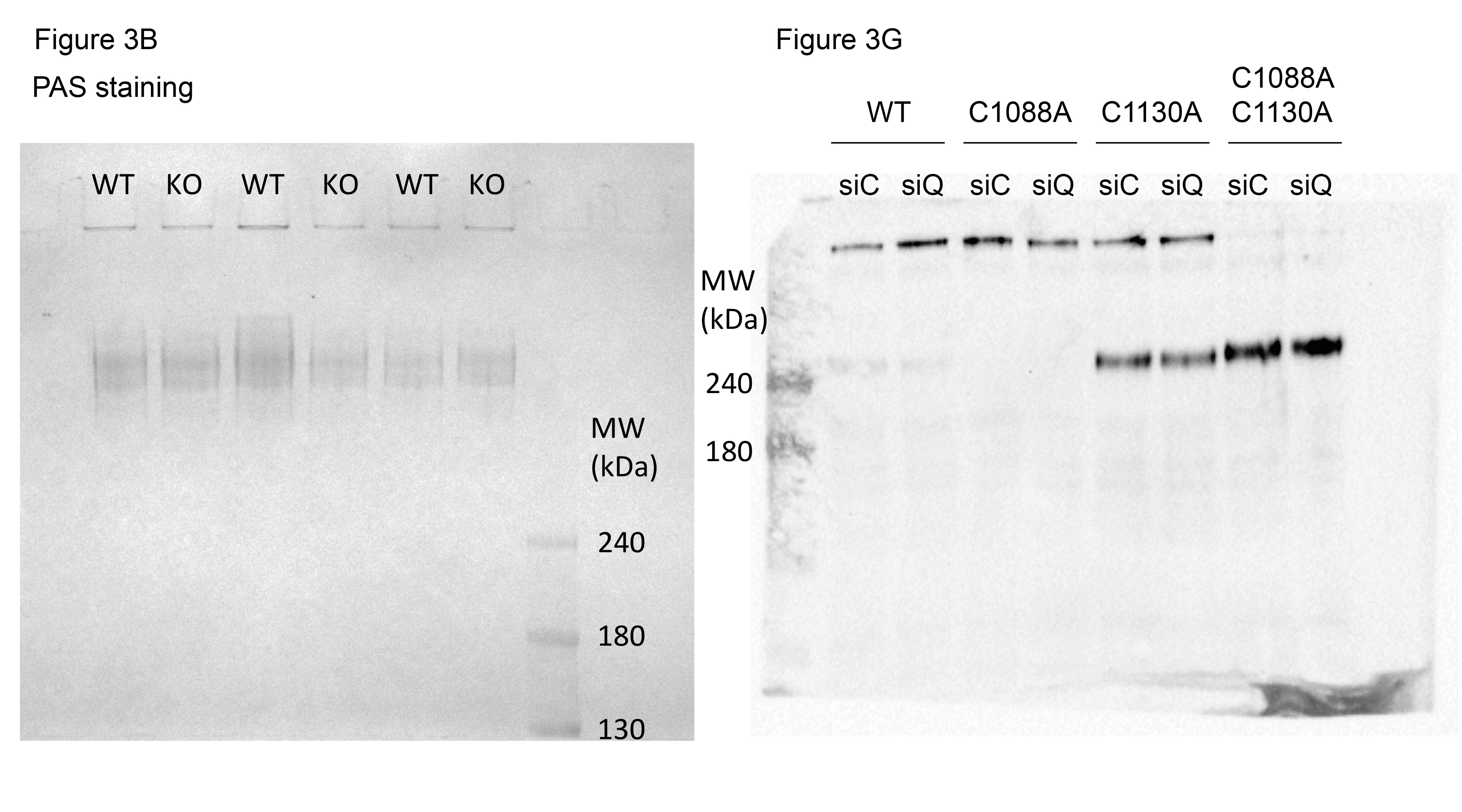

Supplement: Supplementary file 2 — Source Data for Figure 3 [file EMBJ-42-e111869-s003.jpg]

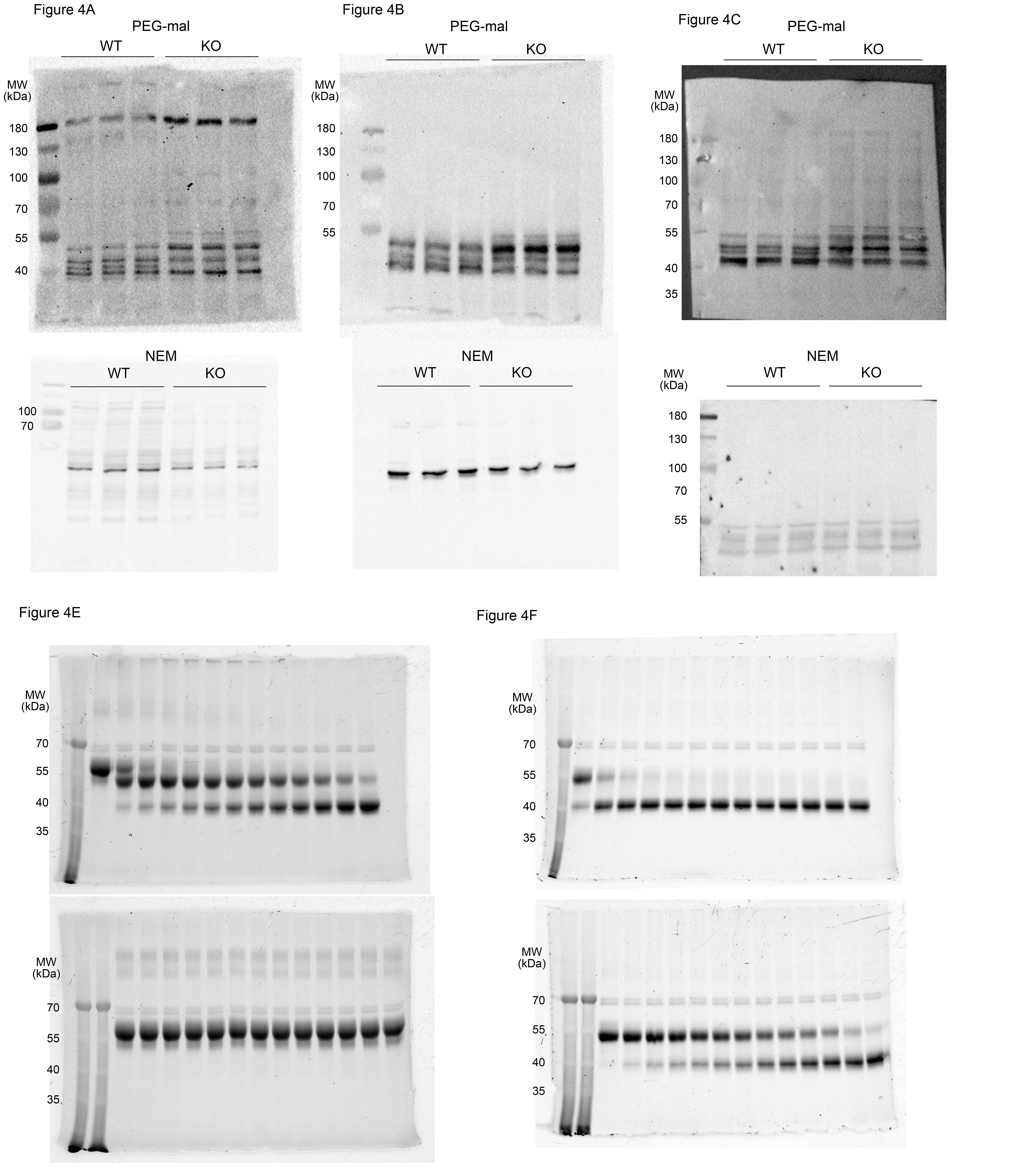

Supplement: Supplementary file 3 — Source Data for Figure 4 [file EMBJ-42-e111869-s002.jpg]
