# Supplementary material for: The disulfide catalyst QSOX1 maintains the colon mucosal barrier by regulating Golgi glycosyltransferases
Source: EMBO J. 2022 Oct 17;42(2):e111869. doi: 10.15252/embj.2022111869 (PMC9841341; doi:10.15252/embj.2022111869)
Supplement: Supplementary file 4 [file EMBJ-42-e111869-s001.pdf]

THE EMBO JOURNAL

The Disulfide Catalyst QSOX1 Maintains the Colon Mucosal Barrier by Regulating Golgi Glycosyltransferases

Deborah Fass, Tal Ilani, Nava Reznik, Noa Yeshaya, Tal Feldman, Patrick Vilela, Zipora Lansky, Gabriel Javitt, Michal Shemesh, Ori Brenner, Yoav Elkis, Neta Varsano, Ana Jaramillo, and Christopher Evans
DOI: 10.15252/embj.2022111869

Corresponding authors: Deborah Fass (deborah.fass@weizmann.ac.il) , Tal Ilani (tal.ilani@weizmann.ac.il)

Review Timeline:

Submission Date:	22nd Jun 22
Editorial Decision:	12th Jul 22
Revision Received:	31st Aug 22
Editorial Decision:	20th Sep 22
Revision Received:	25th Sep 22
Accepted:	29th Sep 22

Editor: Ieva Gailite

Transaction Report:

(Note: With the exception of the correction of typographical or spelling errors that could be a source of ambiguity, letters and reports are not edited. Depending on transfer agreements, referee reports obtained elsewhere may or may not be included in this compilation. Referee reports are anonymous unless the Referee chooses to sign their reports.)

Thank you for submitting your manuscript for consideration by The EMBO Journal. We have now received a full set of referee reports on your manuscript, which are included below for your information.

As you will see from the comments, all reviewers appreciate the quality of your study and the presented role of QSOX1 in regulation of intestinal mucin sialylation, while also indicating some aspects that require further improvement. Based on these positive assessments, I would like to invite you to address the concerns raised by the reviewers in a revised manuscript.

We generally allow three months as standard revision time. As a matter of policy, competing manuscripts published during this period will not negatively impact on our assessment of the conceptual advance presented by your study. However, please contact me as soon as possible upon publication of any related work to discuss the appropriate course of action. Should you foresee a problem in meeting this three-month deadline, please let us know in advance and we may be able to grant an extension.

When preparing your letter of response to the referees' comments, please bear in mind that this will form part of the Review Process File and will therefore be available online to the community. For more details on our Transparent Editorial Process, please visit our website: https://www.embopress.org/page/journal/14602075/authorguide#transparentprocess. Please also see the attached instructions for further guidelines on preparation of the revised manuscript.

Please feel free to contact me if you have any further questions regarding the revision. I would be happy to discuss the revision in more detail via email or phone/videoconferencing.

Thank you for the opportunity to consider your work for publication. I look forward to receiving the revised manuscript.

Referee #1:

This paper investigates the role that QSOX, a powerful oxidase secreted by many cell types and known to impact disulfide bonding of extracellular matrix proteins, plays in mucin biogenesis. Clearly, QSOX-KO mice are hypersensitive to dextran sulphate administration, a treatment known to induce severe damage in gut epithelia.
The authors started their experiments expecting to demonstrate a role of QSOX in the formation of mucin disulfide bonds in post-ER compartments, as in the case of ECM components. Much to their surprise, instead, the experiments revealed that QSOX1 serves to assist the oxidative folding of a set of sialyltransferases, enzymes of pivotal importance in mucin O-

glycosylation in the gut.
Overall, the manuscript is very well written and the figures and panels are well organized and of high quality.

A few points should be taken care of before the manuscript goes into print.

First paragraph: "Single-cell transcriptomics data from large intestine reported by others (Tabula Muris Consortium, 2018) and confirmed by us show that QSOX1 levels are highest in goblet cells": The authors should provide either the reference or the related results.

Figure 1B: the samples WT+IgG and WT+αQSOX1 are not mentioned in the first part of the results (Page 6, subparagraph "QSOX1 KO mice are highly susceptible to induced colitis and have an altered microbiome") but only in the discussion.
The reader might get lost and think that the WT+QSOX1 phenotype should be like the KO one. However, this is not the case. Is it because the inhibitor is against the extracellular QSOX1 and doesn't have an impact on the intracellular enzymes? This is an interesting point that should be clarified.

Figure 1B: The shortening of the colon length in the DSS-induced colitis model is strong evidence of intestinal inflammation. Did the authors observe any colitis-related symptoms differences in KO as compared to the WT mice upon DSS treatment (i.e., diarrhea, bleeding, and body weight loss)?

Figure 1 D: The authors might wish to provide better images of the DSS-treated wt animal-derived colons. From figure 1c, it seems that DSS did not affect that much the crypt structures of the colons, an impairment that, in the DSS model, is usually quite evident. Please explain this unexpected finding or provide more results. It is rather improbable that the colon was not affected at all in WT upon DSS treatment

Figure 1G: Please clarify the conclusion of this result. May the reduction of 16S rRNAs depend on the loss of bacterial species because of the loss of mucus in KO mice? If this were the case, how do the authors explain this complete loss even if they observe an increase of some genera? I would aspect that no differences in the total 16rRNAs occurred.

Figure 3D: the authors state that "both Muc5b and VWF from QSOX1 KO mice polymerized indistinguishably from their counterparts in WT animals" (Page 8, subparagraph "Mucins and related glycoproteins polymerize in QSOX1 KO mice"). However, the results suggest that there are more polymers in KO than in WT mice. Either the sentence should be rephrased or this difference explained.

Figure 3G: in this western blot the authors write that "after depletion of QSOX1, the same extent of dimerization occurred as in control cells" (Page 9, subparagraph "Mucins and related glycoproteins polymerize in QSOX1 KO mice"). Also in this case, there ar more dimers in siQ WT than in siC WT, while the reverse is true for the mutant C1088A. This reviewer agrees that the differences are likely irrelevant, but the harsh statement should be rephrased.

It is not clear why the authors did exclude a role of mucins 1 and 4, especially considering their increase by gene expression profiling (Appendix S2). It would be interesting to learn more about other mucins.

Referee #2:

Fass and coworkers have been interested in the QSOX1 Golgi resident disulfide bond catalyst. This has been assumed to be involved in the late secretory pathway disulfide-bond formation of mucins as QSOX1 is abundant in goblet cells. By using the KO animals, they have now concluded that there is no difference in the assembly of MUC5B and VWF and the functional assumption was wrong. Instead, they had observed the Kellokumpu publication (Hassinen) that showed thar redox levels in cell lines affect glycosylation in an HIF independent way. In this paper, QSOX1 was suggested as one possible mediator. Fass and coworker have now shown that this is correct and that the KO mice have altered glycosylation.

This manuscript has two parts, the first elegantly show that QSOX1 is not involved in mucin or VWF polymerization, an important step forward as this can rule this out. This is important and will direct studies of mucin polymerization along other pathways. The proof obtained from the studies of MUC5B and WVF is convincing.

In the second part they show with lectin staining and simple glycan studies that the QSOX1-KO mice show an altered glycosylation. This is important, especially in the colon where there should normally be an anaerobic milieu for normal commensal bacteria. However, we do not know, not even have an idea, how the host cells could record and control the bacterial composition to make sure that there is an anaerobic milieu. As colon bacteria are selected based on glycans, modulating their composition could be a mechanism. It has also been observed that glycosylation changes with bacterial composition and inflammation. Although this manuscript only give a first glimpse on how this could work, it is a promising beginning.

It should also be pointed out that the Golgi apparatus and how its glycosylation machinery is controlled is an open research area, although for example the already mentioned Kellokumpu have given some insight.

MAJOR

1. The methods say that the animals were littermates, but does not state further how the animal experiments were made. Are the littermate animals kept co-housed? Only 5 animals per cage make it difficult to make sure that the bacteria are not drifting away. Only females?? As this is important, the animal set-ups should also be described in the results and made sure to follow todays standard. The Fig. S1 should be complemented describing which individuals that we kept together.
2. The glycan analyses are made with permethylation that destroys some structures, especially all sulfate groups. The analysis with MALDI-TOF does not discriminate isomers. Thus the glycan analyses are rudimentary. There is no raw data presented. Suggest to limit this part, the glycan differences are clearly shown by lectin staining and the Fig. 5F biosynthetic scheme can be deleted.
3. The 4A-C lanes are either not adding anything or if these are reducing gels (not stated) it is important to show that the total amounts of the enzymes were similar.
4. Page 11, lane 3. PAS stains sugar vicinal diols and varies with the glycan structures. PAS is not a probe for sialic acid. Remove this unnecessary statement.
5. Over all the statistical analyses of differences are lacking.
6. The scale bars are missing on several pictures.

Referee #3:

This is a very well-written and nice manuscript describing the importance of QSOX1 as a catalyst for the formation of disulfide bridges of sialyltransferases involved in the capping of O-glycans in mucins, and in particular in MUC2. The work is highly interdisciplinary and multidisciplinary and encompasses a large number of methodologies to address the function of QSOX1 in biology and in particular in the normal function of MUC2 in the colon. The knockout of QSOX1 reveals how the absence of sialic acid in O-glycans of MUC2 leads to problems in the release of package mucins, shorter colons under DSS treatment, and a decrease in 16S RNA, and even differences in the predominant bacteria species in the colon compared to wild type mice. Overall, the relevance and importance of this manuscript merit publication in EMBO J.

No major changes are suggested to improve the manuscript. However, minor changes that include changes in the text will improve the reading and understanding of the manuscript, see below,

Minor comments:
- In page 4 it is mentioned "...in the second step of mucin....", what is the first step? Define this.
- What is the effect of QSOX1 in other GTs in different pathways or other proteins not related to carbohydrates? It would be nice to add more information on this matter.
- Sentence in page 13 does not read well, "It was clear from the phenotype.....". Change this sentence to improve its readability and understanding.
- The location and presence of STn antigen is becoming controversial nowadays. According to the literature, the STn antigen is acetylated in healthy colon (PMIS:34526666)
and was occasionally observed in healthy gastrointestinal tissue (see PMID: 34526666). Then, a manuscript in 2022 claims that STn is present in normal intestine (PMID: 35303419). What is the antibody used to detect the STn antigen in this manuscript? Does it bind to acetylated STn? In addition, and as the authors might know, the STn antigen is considered a hallmark of numerous types of cancers. Could the authors elaborate more on the type of STn that they detect in their experiments and estimate the degree of its expression?

Response to Reviewers

Referee #1:

This paper investigates the role that QSOX, a powerful oxidase secreted by many cell types and known to impact disulfide bonding of extracellular matrix proteins, plays in mucin biogenesis. Clearly, QSOX-KO mice are hypersensitive to dextran sulphate administration, a treatment known to induce severe damage in gut epithelia.
The authors started their experiments expecting to demonstrate a role of QSOX in the formation of mucin disulfide bonds in post-ER compartments, as in the case of ECM components. Much to their surprise, instead, the experiments revealed that QSOX1 serves to assist the oxidative folding of a set of sialyltransferases, enzymes of pivotal importance in mucin O-glycosylation in the gut.
Overall, the manuscript is very well written and the figures and panels are well organized and of high quality.

A few points should be taken care of before the manuscript goes into print.

First paragraph: "Single-cell transcriptomics data from large intestine reported by others (Tabula Muris Consortium, 2018) and confirmed by us show that QSOX1 levels are highest in goblet cells": The authors should provide either the reference or the related results.

<u>Response</u>
Our results from single-cell transcriptomics showing QSOX1 expression in goblet cells of the colon have been added as Appendix Figure S1.

Figure 1B: the samples WT+IgG and WT+αQSOX1 are not mentioned in the first part of the results (Page 6, subparagraph "QSOX1 KO mice are highly susceptible to induced colitis and have an altered microbiome") but only in the discussion.
The reader might get lost and think that the WT+QSOX1 phenotype should be like the KO one. However, this is not the case. Is it because the inhibitor is against the extracellular QSOX1 and doesn't have an impact on the intracellular enzymes? This is an interesting point that should be clarified.

<u>Response</u>
We added a clarifying sentence to the Results: “Administration of QSOX1 inhibitory antibody (αQSOX1), which inhibits extracellular QSOX1, did not replicate this phenotype (Fig 1B).”
The importance of this experiment is further elaborated in the discussion:
“Importantly, we show here that administration of QSOX1 inhibitory antibodies did not potentiate DSS-induced colitis (Fig 1B), demonstrating that QSOX1 activity in colon physiology, and probably the Golgi functions of QSOX1 in general, are protected from inhibition by systemic inhibitory antibody treatment. Potential use of QSOX1 inhibitory antibodies as cancer therapy would thus not be expected to produce colitis as a side effect.”

Figure 1B: The shortening of the colon length in the DSS-induced colitis model is strong evidence of intestinal inflammation. Did the authors observe any colitis-related symptoms differences in KO as compared to the WT mice upon DSS treatment (i.e., diarrhea, bleeding, and body weight loss)?

Response
We have now added to page 6 the symptoms by which clinical scores were assigned: "QSOX1 KO mice were found to be more sensitive, displaying severe disease symptoms (*i.e.*, diarrhea, rectal bleeding, prolapse) on days four and five, when WT littermates were only mildly affected (*i.e.*, no symptoms or loose stool) (clinical scores were 1-2 for WT and 3-4 for KO, see Methods)."

Figure 1 D: The authors might wish to provide better images of the DSS-treated wt animal-derived colons. From figure 1c, it seems that DSS did not affect that much the crypt structures of the colons, an impairment that, in the DSS model, is usually quite evident. Please explain this unexpected finding or provide more results. It is rather improbable that the colon was not affected at all in WT upon DSS treatment

Response
The relatively low concentration of DSS used in this study (2.5%), and the short time it was administered (5 days), led to very minimal symptoms of colitis in the WT mice. In Okayasu et al., cited as a DSS method reference, mice showed symptoms of colitis within 6–10 days during treatment with 3%–10% DSS. The lack of damage to crypts seen in the colon sections of DSS-treated WT mice in our experiment is consistent with their low clinical scores (1-2). Because this mild treatment produced severe clinical symptoms in the QSOX1 KO mice, the experiment was terminated before the WT mice developed symptoms.

Figure 1G: Please clarify the conclusion of this result. May the reduction of 16S rRNAs depend on the loss of bacterial species because of the loss of mucus in KO mice? If this were the case, how do the authors explain this complete loss even if they observe an increase of some genera? I would aspect that no differences in the total 16rRNAs occurred.

Response
Yes, we definitely think that the reduction in 16S labeling is a result of poor preservation of mucus in the QSOX1 KO colon sections. The microbiome was analyzed from feces samples (as detailed in the methods). The feces likely contain bacteria that are lost along with the unstable mucus in the QSOX1 KO colon sections. This point is made in the "QSOX1 knockout mice have defects in colon mucus" paragraph of the results: "The lack of luminal mucus in fixed *ex vivo* colon samples from QSOX1 KO mice is consistent with the low 16S RNA labeling (Fig 1G), as bacteria inhabit colon mucus."

Figure 3D: the authors state that "both Muc5b and VWF from QSOX1 KO mice polymerized indistinguishably from their counterparts in WT animals" (Page 8, subparagraph "Mucins and related glycoproteins polymerize in QSOX1 KO mice"). However, the results suggest that there

are more polymers in KO than in WT mice. Either the sentence should be rephrased or this difference explained.

<u>Response</u>
This sentence was rephrased according to referee's comment:
"QSOX1 knockout did not prevent polymerization of either Muc5b (Fig 3D) or Vwf (Fig 3E)."

Figure 3G: in this western blot the authors write that "after depletion of QSOX1, the same extent of dimerization occurred as in control cells" (Page 9, subparagraph "Mucins and related glycoproteins polymerize in QSOX1 KO mice"). Also in this case, there ar more dimers in siQ WT than in siC WT, while the reverse is true for the mutant C1088A. This reviewer agrees that the differences are likely irrelevant, but the harsh statement should be rephrased.

<u>Response</u>
We have softened the phrasing to: "similar extents of dimerization occurred as in control cells…"

It is not clear why the authors did exclude a role of mucins 1 and 4, especially considering their increase by gene expression profiling (Appendix S2). It would be interesting to learn more about other mucins.

<u>Response</u>
A QSOX1-dependent role for other mucins in maintaining mucus barrier structure and function is certainly not excluded as a possibility. However, as Muc1 and Muc4 are not known to undergo disulfide-mediated assembly events in the Golgi (and Muc1 lacks extracellular cysteines entirely), we did not focus on these mucins as possible direct targets for QSOX1 sulfhydryl oxidase activity. We were not able to obtain robust immunolabeling of Muc1 or Muc4 in tissue sections, so we could not further evaluate their expression on the protein level. As the decreased Muc2 labeling in KO colons was so striking, and potentially consistent with a barrier defect, we focused on these results, but we agree with the reviewer that it would be interesting to learn whether and how transmembrane mucins contribute to mucsosal barrier function in a redox-dependent manner.

Referee #2:

Fass and coworkers have been interested in the QSOX1 Golgi resident disulfide bond catalyst. This has been assumed to be involved in the late secretory pathway disulfide-bond formation of mucins as QSOX1 is abundant in goblet cells. By using the KO animals, they have now concluded that there is no difference in the assembly of MUC5B and VWF and the functional assumption was wrong. Instead, they had observed the Kellokumpu publication (Hassinen) that showed thar redox levels in cell lines affect glycosylation in an HIF independent way. In this paper, QSOX1 was suggested as one possible mediator. Fass and coworker have now shown that this is correct and that the KO mice have altered glycosylation.

This manuscript has two parts, the first elegantly show that QSOX1 is not involved in mucin or VWF polymerization, an important step forward as this can rule this out. This is important and will direct studies of mucin polymerization along other pathways. The proof obtained from the studies of MUC5B and WVF is convincing.

In the second part they show with lectin staining and simple glycan studies that the QSOX1-KO mice show an altered glycosylation. This is important, especially in the colon where there should normally be an anaerobic milieu for normal commensal bacteria. However, we do not know, not even have an idea, how the host cells could record and control the bacterial composition to make sure that there is an anaerobic milieu. As colon bacteria are selected based on glycans, modulating their composition could be a mechanism. It has also been observed that glycosylation changes with bacterial composition and inflammation. Although this manuscript only give a first glimpse on how this could work, it is a promising beginning.

It should also be pointed out that the Golgi apparatus and how its glycosylation machinery is controlled is an open research area, although for example the already mentioned Kellokumpu have given some insight.

MAJOR

1. The methods say that the animals were littermates, but does not state further how the animal experiments were made. Are the littermate animals kept co-housed? Only 5 animals per cage make it difficult to make sure that the bacteria are not drifting away. Only females?? As this is important, the animal set-ups should also be described in the results and made sure to follow todays standard. The Fig. S1 should be complemented describing which individuals that we kept together.

<u>Response</u>
The issue of co-housing was particularly important for the microbiome experiment. For this experiment, the mice were kept in 2 cages, each containing 3 WT and 3 QSOX1 KO mice. This information was added to methods. The information that WT and KO 1-3 were in one cage, and 4-6 in the other has been added to the legend of what is now Appendix Figure S2. It is specified in the methods section that the induced colitis, microbiome analysis, and transcriptome analysis were done on female mice. The reason for using only females in these experiments was to minimize extraneous variables and because females are more comfortable being co-housed. Aside from the microbiome experiment, littermates were not necessarily co-housed. This information has been added to the methods. For staining and labeling experiments, either male or female mice were analyzed in each experiment, and sex had no effect on the results.

2. The glycan analyses are made with permethylation that destroys some structures, especially all sulfate groups. The analysis with MALDI-TOF does not discriminate isomers. Thus the glycan analyses are rudimentary. There is no raw data presented. Suggest to limit this part, the glycan differences are clearly shown by lectin staining and the Fig. 5F biosynthetic scheme can be deleted.

Response

We have taken the reviewer's suggestion to delete the biosynthetic scheme and glycomics data, and rather to focus on the lectin labelling results.

3. The 4A-C lanes are either not adding anything or if these are reducing gels (not stated) it is important to show that the total amounts of the enzymes were similar.

Response

To emphasize what was added to the lysates in 4A-C, the phrase "as indicated above each blot" was added to the figure legend after, "Lysates were treated with PEG-mal 2 kDa or NEM…" In addition, the following sentence has been added to the legend: "For the experiments in panels A-C, equal amounts of total protein were applied to each gel lane, and no reducing agent was added." The purpose of the control with NEM is now explained more thoroughly in the main text: "As a control showing that the differences in glycosyltransferase migration between WT and KO are due to the addition of PEG-mal, treatment with the small (125 Da) cysteine alkylating agent N-ethylmaleimide (NEM) did not result in appreciable differences in glycosyltransferase migration between WT and KO (Figs 4A-4C)." The following sentence was added to the methods: "Protein concentration in lysates was determined using bicinchoninic acid, and samples containing 30 μg of total protein were separated on SDS-PAGE gels and western blotted for the indicated proteins."

4. Page 11, lane 3. PAS stains sugar vicinal diols and varies with the glycan structures. PAS is not a probe for sialic acid. Remove this unnecessary statement.

Response

We have changed the sentence to: "PAS staining is sensitive to glycan structures and was reported to decrease following sialidase treatment of colon sections (Roe *et al*, 1989)."

5. Over all the statistical analyses of differences are lacking.

Response

We performed statistical analysis of the colon length measurements. Statistical significance indicators were added to the figure, and the following sentence was added to the legend: "Statistical analysis was performed using Tukey multiple comparison of means (* $p<0.05$, ** $p<0.01$)." In addition, p-values for insignificant differences were added to the figure legend: "($p>0.4$ for MAb316.1-treated WT vs. WT control antibody (IgG)-treated; $p>0.5$ for MAb316.1-treated WT vs. WT without antibody treatment)." For microbiome analysis, ANOSIM statistics have been added to the legend of Figure 1E.

6. The scale bars are missing on several pictures.

Response

We apologize that the scale bars had gone missing on Figures 1D and 1G. We added them back.

Referee #3:

This is a very well-written and nice manuscript describing the importance of QSOX1 as a catalyst for the formation of disulfide bridges of sialyltransferases involved in the capping of O-glycans in mucins, and in particular in MUC2. The work is highly interdisciplinary and multidisciplinary and encompasses a large number of methodologies to address the function of QSOX1 in biology and in particular in the normal function of MUC2 in the colon. The knockout of QSOX1 reveals how the absence of sialic acid in O-glycans of MUC2 leads to problems in the release of package mucins, shorter colons under DSS treatment, and a decrease in 16S RNA, and even differences in the predominant bacteria species in the colon compared to wild type mice. Overall, the relevance and importance of this manuscript merit publication in EMBO J.

No major changes are suggested to improve the manuscript. However, minor changes that include changes in the text will improve the reading and understanding of the manuscript, see below,

Minor comments:
- In page 4 it is mentioned "...in the second step of mucin....", what is the first step? Define this.

Response
To clarify, we added the word "First," in our description of the stepwise assembly of mucins: "First, intermolecular disulfide bonds crosslink two mucin carboxy termini to form dimers in the endoplasmic reticulum (ER) (Perez-Vilar *et al*, 1996). Subsequently, multiple mucin dimers are connected to form polymers…"

- What is the effect of QSOX1 in other GTs in different pathways or other proteins not related to carbohydrates? It would be nice to add more information on this matter.

Response
The current Appendix Figure 5 provides examples of proteins with disulfides that are not affected by QSOX1: a glycosyltransferase (Galnt4) that performs an early step in O-linked glycosylation in the Golgi, as well as a non-glycosyltransferase, the PDI family member Pdia4.

- Sentence in page 13 does not read well, "It was clear from the phenotype.....". Change this sentence to improve its readability and understanding.

Response
The paragraph was rearranged to improve logic and readability:
"Mice lacking Muc2 (Van der Sluis *et al*, 2006), Agr2 (Park *et al*, 2009; Zhao *et al*, 2010) or having mutations in Muc2 that interfere with initial mucin folding and assembly (Heazlewood *et al*, 2008) develop spontaneous colitis or display rectal prolapse. QSOX1 KO mice did not display these symptoms, suggesting that early steps in mucin biosynthesis, which occur in the ER,

progress normally in these animals. Based on our observation that colon mucus is compromised in QSOX1 KO mice, it was reasonable to speculate that disulfide-mediated polymerization of mucins in the Golgi apparatus (Perez-Vilar *et al*, 1998) would be defective. It was possible that impaired mucin polymerization led to the secretion of weak and poorly effective mucus, such that the QSOX1 KO mice had relatively normal gut function when unchallenged but were hypersensitive to induced colitis. Despite extensive investigation, however, we were not able to obtain evidence for a mucin disulfide-mediated polymerization defect."

- The location and presence of STn antigen is becoming controversial nowadays. According to the literature, the STn antigen is acetylated in healthy colon (PMIS:34526666)
and was occasionally observed in healthy gastrointestinal tissue (see PMID: 34526666). Then, a manuscript in 2022 claims that STn is present in normal intestine (PMID: 35303419). What is the antibody used to detect the STn antigen in this manuscript? Does it bind to acetylated STn? In addition, and as the authors might know, the STn antigen is considered a hallmark of numerous types of cancers. Could the authors elaborate more on the type of STn that they detect in their experiments and estimate the degree of its expression?

<u>Response</u>
The antibody used in this study is a monoclonal to sialyl Tn (anti STn 219) purchased from Abcam (ab115957). This information is indicated in the Reagent table. This antibody was raised against ovine submaxillary mucin and is reported to react with sialylated Tn antigen, but the sensitivity of this antibody to acetyl masking of the epitope is not specified nor reported. STn is indeed expressed in healthy tissues, while elevated expression is demonstrated in cancer. Colon sections and primary colon cells from QSOX1 KO and WT mice showed similar labeling with the abovementioned STn antibody (Appendix figure 6C and D). Fluorescent labeling cannot provide an estimation to the degree of expression of the immunogen, unless a relevant known quantitative reference is labeled as a control. Therefore, at this point we can only report that labeling with the indicted antibody was not affected by the lack of QSOX1.

Thank you for submitting a revised version of your manuscript. Your study has now been seen by all original referees, who find that their concerns have been addressed and now recommend publication of the manuscript.

There remain a few editorial issues that have to be addressed before I can formally accept your manuscript.

Please let me know if you have any further questions regarding any of these points. You can use the link below to upload the revised files.

Thank you again for giving us the chance to consider your manuscript for The EMBO Journal. I look forward to receiving the final version.

Referee #1:

It is my opinion that this revised paper is now suitable for publication

Referee #2:

No further comment.

Referee #3:

The authors have correctly addressed all my comments, and the manuscript in its current form is suitable for EMBO J.
Therefore, I do not have further comments about this excellent manuscript.

The authors performed the requested editorial changes.

2nd Revision - Editorial Decision 29th Sep 2022

Thank you for addressing the final editorial points. I am now pleased to inform you that your manuscript has been accepted for publication in the EMBO Journal. Congratulations on a great study!

EMBO Press Author Checklist

Corresponding Author Name: Shinji Saiki, Nobutaka hattori	
Journal Submitted to: The EMBO Journal	
Manuscript Number: EMBOJ-2022-111476	

USEFUL LINKS FOR COMPLETING THIS FORM
The EMBO Journal - Author Guidelines
EMBO Reports - Author Guidelines
Molecular Systems Biology - Author Guidelines
EMBO Molecular Medicine - Author Guidelines

Reporting Checklist for Life Science Articles (updated January 2022)
This checklist is adapted from Materials Design Analysis Reporting (MDAR) Checklist for Authors. MDAR establishes a minimum set of requirements in transparent reporting in the life sciences (see Statement of Task: 10.31222/osf.io/9sm4x). Please follow the journal's guidelines in preparing your manuscript.
Please note that a copy of this checklist will be published alongside your article.

Abridged guidelines for figures
1. Data
The data shown in figures should satisfy the following conditions:
- the data were obtained and processed according to the field's best practice and are presented to reflect the results of the experiments in an accurate and unbiased manner.
- ideally, figure panels should include only measurements that are directly comparable to each other and obtained with the same assay.
- plots include clearly labeled error bars for independent experiments and sample sizes. Unless justified, error bars should not be shown for technical replicates.
- if n<5, the individual data points from each experiment should be plotted. Any statistical test employed should be justified.
- Source Data should be included to report the data underlying figures according to the guidelines set out in the authorship guidelines on Data Presentation.

2. Captions
Each figure caption should contain the following information, for each panel where they are relevant:
- a specification of the experimental system investigated (eg cell line, species name).
- the assay(s) and method(s) used to carry out the reported observations and measurements.
- an explicit mention of the biological and chemical entity(ies) that are being measured.
- an explicit mention of the biological and chemical entity(ies) that are altered/varied/perturbed in a controlled manner.
- the exact sample size (n) for each experimental group/condition, given as a number, not a range;
- a description of the sample collection allowing the reader to understand whether the samples represent technical or biological replicates (including how many animals, litters, cultures, etc.).
- a statement of how many times the experiment shown was independently replicated in the laboratory.
- definitions of statistical methods and measures:
  - common tests, such as t-test (please specify whether paired vs. unpaired), simple χ2 tests, Wilcoxon and Mann-Whitney tests, can be unambiguously identified by name only, but more complex techniques should be described in the methods section;
  - are tests one-sided or two-sided?
  - are there adjustments for multiple comparisons?
  - exact statistical test results, e.g., P values = x but not P values < x;
  - definition of 'center values' as median or average;
  - definition of error bars as s.d. or s.e.m.

Please complete ALL of the questions below.
Select "Not Applicable" only when the requested information is not relevant for your study.

Materials

Newly Created Materials	Information included in the manuscript?	In which section is the information available? (Reagents and Tools Table, Materials and Methods, Figures, Data Availability Section)
New materials and reagents need to be available; do any restrictions apply?	Not Applicable	

Antibodies	Information included in the manuscript?	In which section is the information available? (Reagents and Tools Table, Materials and Methods, Figures, Data Availability Section)
For **antibodies** provide the following information:<br>- Commercial antibodies: RRID (if possible) or supplier name, catalogue number and or/clone number<br>- Non-commercial: RRID or citation	Yes	Materials and Methods Section: Western blottin

DNA and RNA sequences	Information included in the manuscript?	In which section is the information available? (Reagents and Tools Table, Materials and Methods, Figures, Data Availability Section)
**Short novel DNA or RNA including primers, probes**: provide the sequences.	Yes	Materials and Methods Section: siRNA and plasmid transfection, Quantitative RT-PCR

Cell materials	Information included in the manuscript?	In which section is the information available? (Reagents and Tools Table, Materials and Methods, Figures, Data Availability Section)
**Cell lines:** Provide species information, strain. Provide accession number in repository **OR** supplier name, catalog number, clone number, and/**OR** RRID.	Yes	Materials and Methods Section: Cell cultre
**Primary cultures:** Provide species, strain, sex of origin, genetic modification status.	Not Applicable	
Report if the cell lines were recently **authenticated** (e.g., by STR profiling) and tested for mycoplasma contamination.	Not Applicable	

Experimental animals	Information included in the manuscript?	In which section is the information available? (Reagents and Tools Table, Materials and Methods, Figures, Data Availability Section)
**Laboratory animals or Model organisms:** Provide species, strain, sex, age, genetic modification status. Provide accession number in repository **OR** supplier name, catalog number, clone number, **OR** RRID.	Not Applicable	
**Animal observed in or captured from the field:** Provide species, sex, and age where possible.	Not Applicable	
Please detail **housing and husbandry conditions**.	Not Applicable	

Plants and microbes	Information included in the manuscript?	In which section is the information available? (Reagents and Tools Table, Materials and Methods, Figures, Data Availability Section)
**Plants:** provide species and strain, ecotype and cultivar where relevant, unique accession number if available, and source (including location for collected wild specimens).	Not Applicable	
**Microbes:** provide species and strain, unique accession number if available, and source.	Not Applicable	

Human research participants	Information included in the manuscript?	In which section is the information available? (Reagents and Tools Table, Materials and Methods, Figures, Data Availability Section)
If collected and within the bounds of privacy constraints report on age, sex and gender or ethnicity for all study participants.	Yes	Table S1

Core facilities	Information included in the manuscript?	In which section is the information available? (Reagents and Tools Table, Materials and Methods, Figures, Data Availability Section)
If your work benefited from core facilities, was their service mentioned in the acknowledgments section?	Yes	Acknowledgement

Design

Study protocol	Information included in the manuscript?	In which section is the information available? (Reagents and Tools Table, Materials and Methods, Figures, Data Availability Section)
If study protocol has been **pre-registered, provide DOI in the manuscript**. For clinical trials, provide the trial registration number **OR** cite DOI.	Not Applicable	
Report the **clinical trial registration number** (at ClinicalTrials.gov or equivalent), where applicable.	Not Applicable	

Laboratory protocol	Information included in the manuscript?	In which section is the information available? (Reagents and Tools Table, Materials and Methods, Figures, Data Availability Section)
Provide DOI OR other citation details if **external detailed step-by-step protocols** are available.	Not Applicable	

Experimental study design and statistics	Information included in the manuscript?	In which section is the information available? (Reagents and Tools Table, Materials and Methods, Figures, Data Availability Section)
Include a statement about **sample size** estimate even if no statistical methods were used.	Yes	Figure legend of each graph.
Were any steps taken to minimize the effects of subjective bias when allocating animals/samples to treatment (e.g. **randomization procedure**)? If yes, have they been described?	Not Applicable	
Include a statement about **blinding** even if no blinding was done.	Not Applicable	
Describe **inclusion/exclusion criteria** if samples or animals were excluded from the analysis. Were the criteria pre-established?<br><br>If sample or data points were omitted from analysis, report if this was due to attrition or intentional exclusion and provide justification.	Not Applicable	
For every figure, are **statistical tests** justified as appropriate? Do the data meet the assumptions of the tests (e.g., normal distribution)? Describe any methods used to assess it. Is there an estimate of variation within each group of data? Is the variance similar between the groups that are being statistically compared?	Yes	Figure legends.

Sample definition and in-laboratory replication	Information included in the manuscript?	In which section is the information available? (Reagents and Tools Table, Materials and Methods, Figures, Data Availability Section)
In the figure legends: state number of times the experiment was **replicated** in laboratory.	Yes	Figure legends.
In the figure legends: define whether data describe **technical or biological replicates**.	Yes	Figure legends.

Ethics

Ethics	Information included in the manuscript?	In which section is the information available? (Reagents and Tools Table, Materials and Methods, Figures, Data Availability Section)
Studies involving **human participants**: State details of **authority granting ethics approval** (IRB or equivalent committee(s), provide reference number for approval.	Yes	Materials and Methods Section: Ethics statement
Studies involving **human participants**: Include a statement confirming that **informed consent** was obtained from all subjects and that the experiments conformed to the principles set out in the WMA Declaration of Helsinki and the Department of Health and Human Services Belmont Report.	Yes	Materials and Methods Section: Ethics statement
Studies involving **human participants:** For publication of **patient photos**, include a statement confirming that consent to publish was obtained.	Not Applicable	
Studies involving experimental **animals**: State details of **authority granting ethics approval** (IRB or equivalent committee(s), provide reference number for approval. Include a statement of compliance with ethical regulations.	Not Applicable	
Studies involving **specimen and field samples:** State if relevant **permits** obtained, provide details of authority approving study; if none were required, explain why.	Not Applicable	

Dual Use Research of Concern (DURC)	Information included in the manuscript?	In which section is the information available? (Reagents and Tools Table, Materials and Methods, Figures, Data Availability Section)
Could your study fall under dual use research restrictions? Please check biosecurity documents and list of **select agents and toxins** (CDC): https://www.selectagents.gov/sat/list.htm	Not Applicable	
If you used a select agent, is the security level of the lab appropriate and reported in the manuscript?	Not Applicable	
If a study is subject to dual use research of concern regulations, is the name of the **authority granting approval and reference number** for the regulatory approval provided in the manuscript?	Not Applicable	

Reporting

The MDAR framework recommends adoption of discipline-specific guidelines, established and endorsed through community initiatives. Journals have their own policy about requiring specific guidelines and recommendations to complement MDAR.

Adherence to community standards	Information included in the manuscript?	In which section is the information available? (Reagents and Tools Table, Materials and Methods, Figures, Data Availability Section)
State if relevant guidelines or checklists (e.g., **ICMJE, MIBBI, ARRIVE, PRISMA**) have been followed or provided.	Not Applicable	
For **tumor marker prognostic studies,** we recommend that you follow the **REMARK** reporting guidelines (see link list at top right). See author guidelines, under 'Reporting Guidelines'. Please confirm you have followed these guidelines.	Not Applicable	
For **phase II and III randomized controlled trials**, please refer to the **CONSORT** flow diagram (see link list at top right) and submit the CONSORT checklist (see link list at top right) with your submission. See author guidelines, under 'Reporting Guidelines'. Please confirm you have submitted this list.	Not Applicable	

Data Availability

Data availability	Information included in the manuscript?	In which section is the information available? (Reagents and Tools Table, Materials and Methods, Figures, Data Availability Section)
Have **primary datasets** been deposited according to the journal's guidelines (see 'Data Deposition' section) and the respective accession numbers provided in the Data Availability Section?	Yes	Materials and Methods section: Data availability
Were **human clinical and genomic datasets** deposited in a public access-controlled repository in accordance to ethical obligations to the patients and to the applicable consent agreement?	Not Applicable	
Are **computational models** that are central and integral to a study available without restrictions in a machine-readable form? Were the relevant accession numbers or links provided?	Not Applicable	
If publicly available data were reused, provide the respective **data citations in the reference list**.	Not Applicable	
